# Distinct SARS-CoV-2 antibody reactivity patterns elicited by natural infection and mRNA vaccination

**DOI:** 10.1038/s41541-021-00396-3

**Published:** 2021-11-04

**Authors:** Rafael Assis, Aarti Jain, Rie Nakajima, Algis Jasinskas, Saahir Khan, Anton Palma, Daniel M. Parker, Anthony Chau, Specimen Collection Group, Sina Hosseinian, Milind Vasudev, Connie Au, Kathleen Powers, Paramveer S. Birring, Brandon Chin, Rana Andary, Joshua M. Obiero, Delia Tifrea, Amanda Leung, Christina Grabar, Fjolla Muqolli, Ghali Khalil, Jessica Colin Escobar, Jenny Ventura, D. Huw Davies, Bruce Albala, Bernadette Boden-Albala, Sebastian Schubl, Philip L. Felgner

**Affiliations:** 1 School of Medicine and the Vaccine R&D Center, University of California Irvine, Irvine, CA USA; 2 Division of Infectious Diseases, University of Southern California, Los Angeles, CA USA; 3 Institute for Clinical & Translational Science, University of California Irvine, Irvine, CA USA; 4 Department of Population Health & Disease Prevention, Program in Public Health, University of California Irvine, Irvine, CA USA; 5 Department of Statistics, University of California Irvine, Irvine, CA USA; 6 School of Medicine, University of California Irvine, Irvine, CA USA; 7 Department of Surgery, School of Medicine, University of California Irvine, Irvine, CA USA

**Keywords:** Antibodies, RNA vaccines

## Abstract

We analyzed data from two ongoing COVID-19 longitudinal serological surveys in Orange County, CA., between April 2020 and March 2021. A total of 8476 finger stick blood specimens were collected before and after a vaccination campaign. IgG levels were determined using a multiplex antigen microarray containing antigens from SARS-CoV-2, SARS, MERS, Common CoV, and Influenza. Twenty-six percent of specimens from unvaccinated Orange County residents in December 2020 were SARS-CoV-2 seropositive; out of 852 seropositive individuals 77 had symptoms and 9 sought medical care. The antibody response was predominantly against nucleocapsid (NP), full length, and S2 domain of spike. Anti-receptor binding domain (RBD) reactivity was low and not cross-reactive against SARS S1 or SARS RBD. A vaccination campaign at the University of California Irvine Medical Center (UCIMC) started on December, 2020 and 6724 healthcare workers were vaccinated within 3 weeks. Seroprevalence increased from 13% pre-vaccination to 79% post-vaccination in January, 93% in February, and 99% in March. mRNA vaccination induced higher antibody levels than natural exposure, especially against the RBD domain and cross-reactivity against SARS RBD and S1 was observed. Nucleocapsid protein antibodies can be used to distinguish vaccinees to classify pre-exposure to SARS-CoV-2 Previously infected individuals developed higher antibody titers to the vaccine than non pre-exposed individuals. Hospitalized patients in intensive care with severe disease reach significantly higher antibody levels than mild cases, but lower antibody levels compared to the vaccine. These results indicate that mRNA vaccination rapidly induces a much stronger and broader antibody response than SARS-CoV-2 infection.

## Introduction

Protective efficacy of SARS-CoV-2 spike mRNA vaccines reported by the developers, Pfizer and Moderna, has been successful, showing convincing evidence of protection as short as 14 days after the first immunization^1,2^. This timeframe is similar to the observed seroconversion times of natural infection that ranges from 10 to 14 days^3,4^. However, in contrast to the vaccine, it is not yet clear how protective the antibodies induced by natural infection are and how long the protection will last as reports have shown that antibodies generated in response to the infection wane after a few months and can reach baseline levels before the first year^4^.

**Fig. 1:**
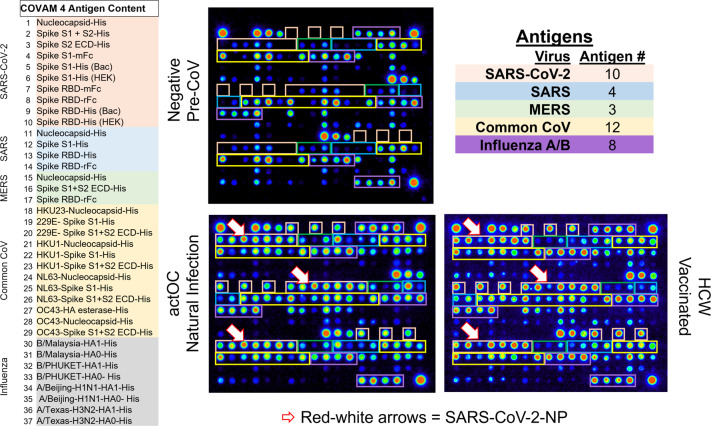
Coronavirus antigen microarray—COVAM. The content of the coronavirus antigen microarray is shown. There are 10 SARS-CoV-2 antigens, 3 SARS, 3 MERS, 12 Common COV, and 8 influenza antigens. Each antigen is printed in triplicate and organized as shown on the images with Orange boxes around the SARS-CoV-2 antigens, Blue SARS, Green MERS, Yellow Common CoV, and Purple for Influenza. Three different samples are shown, a negative Pre-CoV, natural infection (actOC), and a sample from an mRNA vaccinee (HCW). The Pre-CoV sample has negligible reactivities to SARS-CoV-2, SARS, and MERS, whereas natural infection and the vaccinees have significant antibodies against the novel CoV. The red-white arrows point to the nucleocapsid protein which detects antibodies in naturally exposed people but not in the vaccinees.

To further understand the mRNA vaccine induced immune response we were interested to compare the antibody response induced by the vaccine with that induced by natural exposure to SARS-CoV-2. Here we show results using a multiplex solid phase immunofluorescent assay for quantification of human antibodies against 37 antigens from SARS-CoV-2, other novel and common coronaviruses, and influenza viruses that are causes of respiratory infections (Fig. 1)^5–9^. This coronavirus antigen microarray (COVAM) assay uses a small volume of blood derived from a finger stick, does not require the handling of infectious virus, quantifies the level of different antibody types in serum and plasma and is amenable to scaling-up. Finger stick blood collection enables large scale epidemiological studies to define the risk of exposure to SARS-CoV-2 in different settings^10^. Since the assay requires 1 microliter of blood it is also practical for monitoring immunogenicity in neonates, children, and small animal models.

The concept of nucleic acid vaccines appeared 30 years ago after it was shown that intramuscular (IM) injection of a plasmid encoding HIV gp120 was able express the transgene at the site of injection and induce the production of anti-gp120 antibody^11^. That was followed by a 1993 report showing the efficacy of an influenza nucleic acid vaccine in a rodent model^12^. This was a nucleocapsid based nucleic acid vaccine that induced cross-subtype protection against both group 1 and group 2 viruses (A/PR/8/34 (H1N1) and A/HK/68 (H3N2)).

The utility of cationic lipids for gene delivery was discovered and reported in 1987^13^ and synthetic self-assembling lipoplexes for gene delivery described^14–16^. These technologies spawned a branch of gene therapy science, and an NIH study section, Genes and Drug Delivery (GDD) was established in 2002. Since then, synthetic gene delivery system research and nucleic acid vaccine science has flourished. DNA vaccines were the first nucleic acid vaccines to be manufactured and tested on a pharmaceutical scale^17,18^. The mRNA vaccines that are being distributed so widely today may seem to have suddenly emerged, but there has been 30 years of scientific discovery, discourse and development, work from hundreds of scientists, numerous biotechnology companies and billions of public and private dollars invested enabling this effective response with a COVID mRNA vaccine at this moment.

Our results show that mRNA vaccines are remarkably effective at elevating antibody levels against SARS-CoV-2 antigens, rapidly converting seronegative individuals to seropositive. The observed seroconversion level and breadth across diverse coronavirus strains induced by the mRNA vaccines is much greater than that induced by natural infection. After probing more than 8,729 pre- and post-vaccination specimens our results confirm that the mRNA vaccines can be used effectively in a vaccination campaign to immunize large groups within a matter of weeks.

## Results

### mRNA vaccination achieves 99% seropositivity within 3 months after initiating an intensive vaccination campaign

**Fig. 2:**
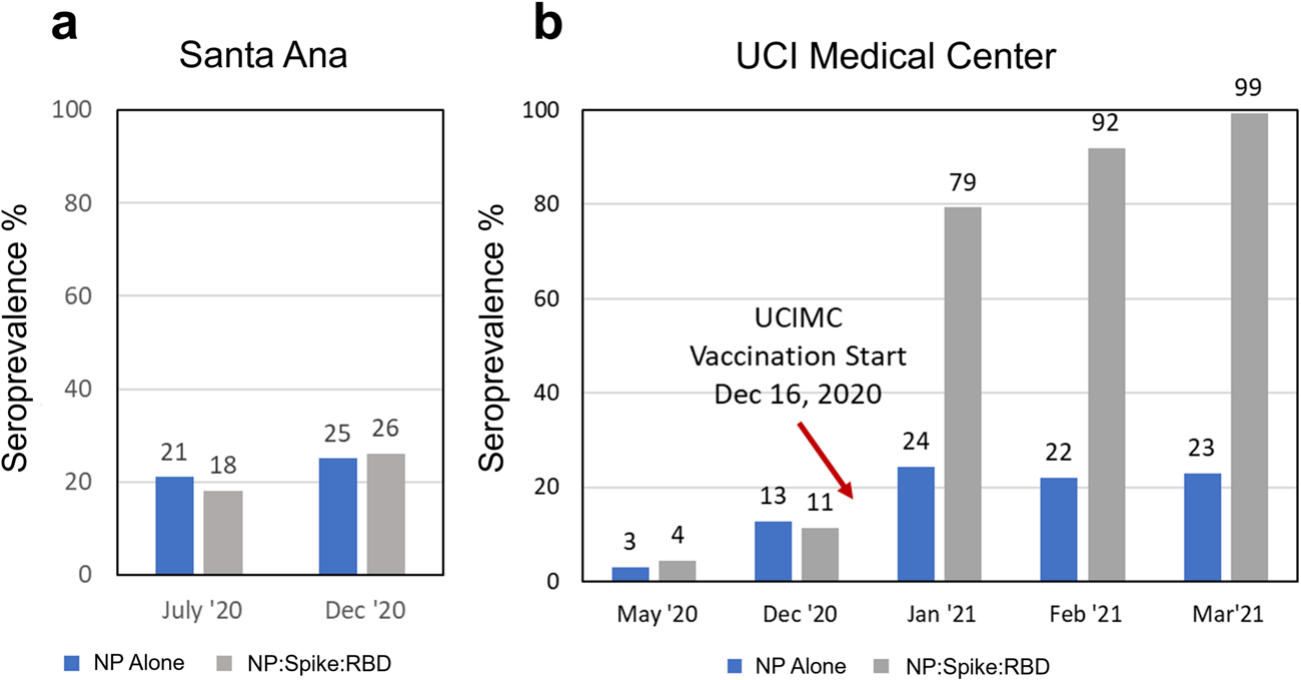
Coronavirus seroprevalence of Naturally exposed and Vaccinated populations. **a** Finger stick blood specimens were collected from Orange County in July (2979 specimens) and Santa Ana in December (3347 specimens), and seroprevalence measured on the COVAM array. **b** Seroprevalence in cross-sections from the UCI Medical Center was measured by COVAM analysis in May and December before the start of the mRNA vaccination campaign on December 16, 2020 and monthly post vaccination time points in 2021. The gray bar is the COVAM seroprevalence prediction and the blue bar is the nucleocapsid protein seropositivity. The graph shows the increase in reactivity to Spike-RBD in relation to the nucleoprotein in the vaccination population reaching a seropositivity of 99% as opposed to 23% (for the NP). For the Santa Ana population, an increase in seroprevalence was observed, but no differential increase for Spike-RBD was observed.

Beginning in March 2020 this study was designed to track the seroprevalence at UCIMC healthcare workers (HCW) and the Orange County community residents during the outbreak. (Table 1). In July the observed seroprevalence in Santa Ana zip codes was 18%, and in December it increased to 26% (Fig. 2a). Prior to the vaccination campaign in December 2020, the seroprevalence at the UCIMC reached 13%, half of the prevalence level measured in Santa Ana. This observation suggests that strict transmission control measures enforced at the hospital played a role in keeping COVID-19 exposure levels low. On December 16, 2020 the vaccination campaign started at the hospital and seroprevalence for the UCIMC HCW population jumped from 13% (early December) to 78% in January, to 93% in February, and 98.7% in the last week of March 2021 (Fig. 2b). This observation strongly corroborates the high efficacy of the nucleic acid vaccine in stimulating an antibody response and also highlights the success of the vaccination campaign that immunized 6724 HCW from 12/16/2020 to 1/05/2021, and 10,000 more since then.

**Table 1:** Longitudinal study design, sample collection, and assay parameters.

Samples tested			Measurements	
Collection	Number	Date	Virus	Antigen #
Orange County	2979	July ’20	SARS-CoV-2	10
Santa Ana	3347	Dec ‘20	SARS	4
UCI Healthcare Workers	1060	May ‘20	MERS	3
UCI Healthcare Workers	313	Dec ‘20	Common CoV	12
			Influenza A/B	8
Vaccination Start Date	December 16, 2020		Total	37
UCI Healthcare Workers	140	Jan ‘21	Triplicate	111
UCI Healthcare Workers	750	Feb ‘21	IgG&IgM	222
UCI Healthcare Workers	157	Mar ‘21		
Total	8746		Specimens	8746
			Measurement#	1,941,612

Study Design. Finger stick blood specimens were collected at weekly intervals from drive-through locations around Orange County and from healthcare workers at the University of California Medical Center. Individual samples were probed on the COVAM, quantified and analyzed. Personalized serology reports were generated and linked to individual QR codes for everyone to access their own report.

**Fig. 3:**
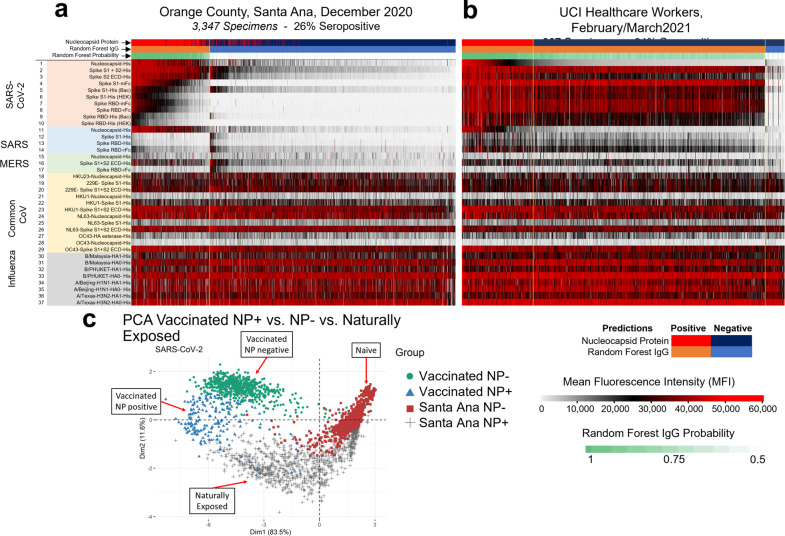
Antibody reactivity of the Santa Ana and health care workers groups. The heat maps show all of the IgG reactivity data from 3347 pre-vaccination specimens collected from Santa Ana in December 2020 (**a**), and 907 post-vaccination specimens collected from the UCIMC in February (**b**). The 37 antigens are in rows and the specimens are in 3347 columns for (**a**) and 907 columns for (**b**). The level of antibody measured in each specimen against each antigen is recorded as mean fluorescence intensity (MFI) according to the graduated scale from 0 to 60,000. Red is a high level, white a low level and black is in between. **a** Samples are classified as either SARS-CoV-2 seropositive clustered to the left (orange bar) or seronegative and clustered to the right (blue bar). Seropositive specimens recognize nucleoprotein and full-length spike. RBD segments are recognized less well. **b** Reactivity of specimens from 907 UCIMC HCW, 94% were vaccinated and seropositive. The heatmap shows that seropositive vaccinees in the HCW cohort can be classified into two groups, either seropositive for nucleoprotein or not, whereas the naturally exposed population (**a**) is uniformly seropositive for both nucleoprotein and full-length spike. **c** Principal component analysis of the protein microarray data in this study. The specimens fall into 4 distinct groups based on their reactivity against 10 SARS-CoV-2 antigens. Naturally exposed individual separate from unexposed naives, the naturally exposed separate from the vaccinees, and the vaccinees separate into 2 groups depending on whether they are seropositive for NP or not.

**Fig. 4:**
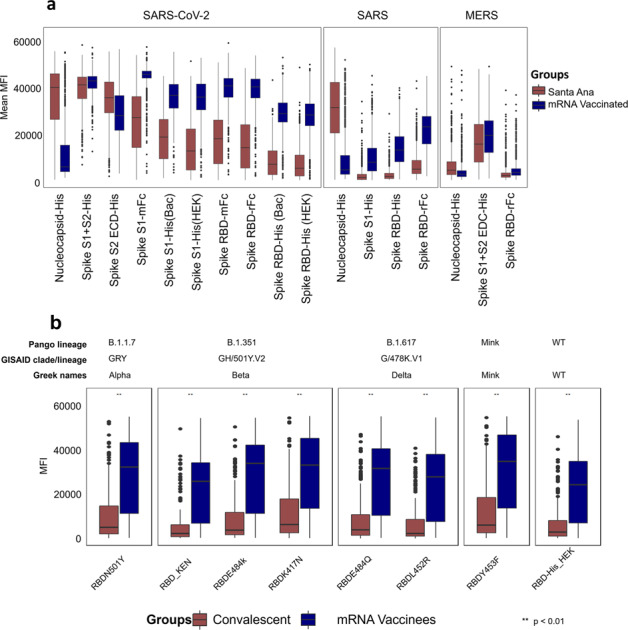
Natural exposure vs mRNA vaccination antibody reactivity. Mean MFI signals for each of the novel coronavirus antigens in the natural exposure cohort from Santa Ana in December 2020 in brown and the February/March 2021 vaccination group (in blue) are plotted. The boxes represent the first quartile, median, and, third quartile and the whiskers extend 1.5 times the interquartile range (IQR). Wilcoxon test was performed for pairwise comparisons and *p* values lower than 0.01 were considered significant and represented as **. Panel **a** shows that antibody responses against Spike RBD variants are significantly elevated in mRNA vaccinated people compared to naturally exposed individuals. The **b** shows that antibody responses against RBD variants from the Coronavirus Pango Lineages B1.1.7, B.1.351, B.1.617, Minkvariant, and the Wild Type. In blue, are individuals that were immunized with a SARS-CoV-2 vaccine based on an adenovirus vector and in brown, convalescent individuals. As shown, mRNA vaccinees display a stronger reactivity to all variants when compared to the other two groups.

Differences were noted in the antibody responses induced by the vaccine compared to natural exposure. (Fig. 2). The nucleocapsid protein is an immunodominant antigen for which the antibody response increases in concordance with natural exposure (Figs. 2a, 3a, and 4). However, nucleocapsid is not a component of the mRNA vaccines and consequently there is no vaccine induced increase in antibodies against this antigen. Accordingly, anti-spike antibody levels increased in vaccinees while the nucleocapsid protein antibody level remained constant between Jan and March 2021. (Fig. 2b) This suggested that anti-nucleocapsid antibodies can be used as a biomarker of prior natural exposure within a population of seropositive vaccinees.

### Natural exposure and mRNA induced antibody profiles and the anti-nucleocapsid antibody biomarker of natural exposure

Data from 3347 specimens collected from Santa Ana residents in December 2020 are shown in the heatmap Fig. 3a. The level of antibody measured in each specimen against each antigen is recorded as mean fluorescence intensity (MFI) according to the graduated scale from 0 to 60,000. In order to assess the seroreactivity, we utilized a random forest based prediction algorithm that used data from a well characterized training set (pre-CoV seronegatives collected in 2019 and PCR-confirmed positive cases) to classify the samples as seroreactive or not seroreactive^6,7^. This algorithm was constructed to classify SARS-CoV-2 serostatus using reactivity of 10 SARS-CoV-2 antigens to maximize sensitivity and specificity. With this machine learning algorithm, the samples were classified as either SARS-CoV-2 seropositive, grouped to the left, or seronegative and clustered to the right (Fig. 3a). Seropositive specimens recognize nucleoprotein and full-length spike. RBD segments are recognized less well.

The heatmap in Fig. 3b shows the reactivity of specimens from 907 UCIMC healthcare workers collected in February and March after the vaccination campaign.; 93.8% were seropositive, of whom most were vaccinated. The anti-SARS-CoV-2 antibody reactivity induced by vaccination (Fig. 3b) differs from the antibody profile induced by natural exposure (Fig. 3a). The vaccine induces higher antibody levels against the RBD containing segments compared to the level induced by natural exposure in the Santa Ana cohort.

Since all adults in these cohorts are exposed to seasonal colds, influenza virus infections, and influenza vaccinations, all the individuals have baseline antibody levels against common-cold CoV and influenza. Thus, background antibody levels against common CoV and influenza antigens are elevated in both the Santa Ana and HCW groups irrespective of whether they are COVID seropositive or not.

Principal component analysis (PCA) using the reactivity to the SARS-CoV-2 antigens (Fig. 3c) shows that seroreactive samples from the two study groups fall into two clusters (mainly along the 1st dimension axis) indicating that the antibody response to the vaccine differs from the antibody response induced by natural infection. Naturally exposed individuals are distinct from the naive SARS-CoV-2 seronegative individuals. In addition, the heatmap (Fig. 3c) clusters seropositive vaccinees into two groups based on whether they are seropositive for SARS-CoV-2 NP or not. The naturally exposed population (Fig. 3) shows high reactivity to both SARS-CoV-2 NP and full-length spike (S1 + S2). This is also evident in the PCA analysis which shows distinct clustering according to the reactivity to the nucleocapsid protein (NP, mainly along the dimension 2 axis).

### mRNA vaccines induce higher antibody levels and greater antibody breadth than natural exposure to infection

Mean MFI signals for each of the novel coronavirus antigens in the Santa Ana natural exposure and the UCIMC vaccination healthcare workers groups are plotted in Fig. 4a. Natural exposure in seropositive people induces high antibody levels against NP, full-length spike (S1 + S2) and the S2 domain. Antibodies against S1 and the RBD domains are lower. Vaccinated individuals have high antibody levels against full-length spike and the S2 domain of SARS-CoV-2 spike, and significantly higher antibody levels against S1 and the RBD domains compared to naturally exposed individuals. In natural exposure there was no significant cross-reactivity against SARS S1 or the RBD domains. Surprisingly, the vaccine induced significant cross-reactive antibodies against the SARS spike and SARS RBD. Cross-reactivity against SARS NP and full-length MERS S protein is evident in both the natural exposure and vaccinated groups.

A protein microarray was printed containing 8 variant RBD domains from the alpha, beta, delta, mink and wild type linages. The array was probed with a collection of convalescent plasma form people who recovered from a confirmed COVID-19 case and compared with plasma from the mRNA vaccinees. The mRNA vaccinees induced significantly higher antibody levels against each of the RBD variants compared to the levels induced after infection in convalescent plasma (Fig. 4b).

### mRNA vaccination induces higher antibody levels than severe acute infection

The differences between the mRNA vaccinated and Santa Ana groups could be due to relatively low exposure in this naturally exposed population. The results in Table 2 remarkably show that in the group of 852 COVID seropositive individuals only 38 had a confirmatory COVID-19 positive PCR test and only 9 of those sought medical care for symptoms. These results indicate that the majority of SARS-CoV-2 exposures in the Santa Ana community are mild, asymptomatic, and unreported. We wondered whether low anti-RBD reactivity levels observed in this cohort is because the cases were mild. Indeed, we have reported that that elevated levels of antibody against a SARS-CoV-2 NP epitope predicts disease severity in COVID-19 patients^19^. Consequently, we compared the antibody levels among 93 hospitalized individuals who were either in intensive care or not and the results are shown in Supplementary Fig. 5.

**Table 2:** Symptom descriptive in December and percent positivity.

Descriptive symptom	COVID Seropositive
	(*N* = 852)
I had symptom(s)	77 (9%)
I was tested for COVID-19	128 (15%)
I had a positive COVID-19 test	38 (4%)
I sought medical care for symptoms	7 (1%)
I was hospitalized because of these symptoms	2 (0.2%)
I was admitted to the ICU because of these symptoms	0

The UCIMC biorepository is a collection of 563 longitudinal plasma specimens collected from 93 patients who experienced an extended hospital stay while recovering from COVID. A subset of patients was admitted to the intensive care unit on ventilator and classified as severe, and the remaining patients with milder disease did not require ICU. The results in Supplementary Fig. 5 show that the low anti-RBD response in the Santa Ana residents is equivalent to the antibody response induced in qPCR confirmed, symptomatic, acutely infected hospitalized patients with relatively mild disease. There is no significant level of cross reactivity against SARS in this group. The anti-RBD response measured in the severe ICU patients is significantly elevated compared to the milder cases and the group of largely asymptomatic individuals from Santa Ana. Severe cases have significant cross reactivity against the SARS RBD domains. However, the severe UCI patients do not reach the level of anti-RBD reactivity as mRNA vaccinated individuals. Vaccination induces a more robust and cross reactive antibody response than natural exposure alone, suggesting that those who have recovered from COVID benefit from the vaccination with stronger and broader antibody response. The complete list of *p*-values can be found in Supplementary Table 2.

### mRNA vaccines induce antibodies that cross-react against SARS spike

Cross-reactivity of the SARS-CoV-2 NP antibodies induced by exposure to the virus, against NP from SARS is evident from the scatterplot in Supplementary Fig. 4A. The antibodies induced by SARS-CoV-2 infection react equally against NP from both SARS-CoV-2 and SARS. Cross-reactivity against SARS NP and full-length MERS full length spike protein is also evident in both the natural exposure and vaccinated groups. (Fig. 4a) However, significant cross-reactivity to SARS S1 and SARS RBD domains was only observed in the mRNA vaccine group.

From a large group of specimens, we can use the *R*^2^ value as a measure of cross reactivity comparing any 2 antigens that are printed on the array. When two antigens produce the same signals for each specimen it is evidence of cross reactivity. For example, the scatterplot comparing nucleocapsid proteins (NP) from SARS-CoV-2 and SARS gives *R*^2^ = 0.93 and a slope of 1.1, indicating that the antibodies produced after SARS-CoV-2 infection cross-react against SARS NP. (Supplementary Fig. 4A) A similar plot comparing infection induced antibody levels against SARS-CoV-2 S1 do not correlate with antibodies against hCoV-299E S1 (*R*^2^ = 0.009), indicating no cross reactivity against this common cold antigen.

**Fig. 5:**
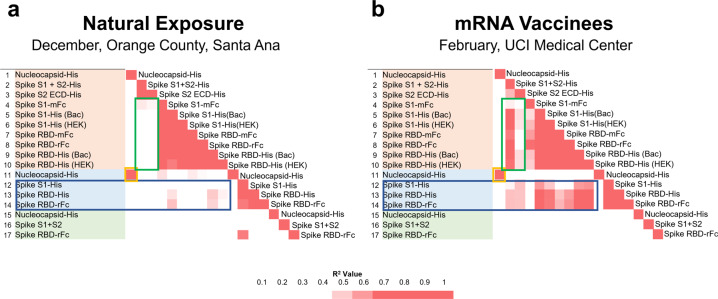
COVAM pairwise correlation matrices. Correlation matrices with all pairwise comparisons between all antigens on the COVAM array were generated. The heatmaps represent a color scale of the r-squared of each pairwise comparison. On **a** is shown the correlation matrix for the Orange County group (actOC Natural exposure) and in **b** is shown the UCIMC vaccinated group. The mRNA vaccine induces cross reactive antibodies against SARS S1 and the RBDs (**b**, Blue Box) and natural exposure does not (**a**) Similarly, vaccine induced antibodies against full length spike cross-react with SARS-CoV-2 RBD (**b**, Green Box) and the natural exposure does not (**a**).

There are 37 antigens on the COVAM and 702 pairwise comparisons. The *R*^2^ values for all pairwise comparisons are plotted on the correlation matrices in Fig. 5. Figure 5a plots cross-reactivity of antibodies induced by natural exposure, and Fig. 5b the cross-reactivity of antibodies induced by vaccination, the complete matrices are available in the Supplementary Table 3. Natural exposure induces SARS-CoV-2 NP antibodies that cross react with SARS NP (Orange Box). Antibodies against full length spike cross reacts with the S2 domain but not against the S1 Domain or RBD. (Fig. 5a, Green box). Antibodies against the S2 domain do not cross react against the S1 domain or RBD. Full length spike antibodies induced after infection are well correlated with the S2 domain but no significant cross reactivity evident against SARS S1 or SARS RBD (Fig. 5a, Blue box). mRNA vaccination (Fig. 5b) shares cross-reactivity of natural exposure and also induces antibodies against full length spike that cross-reacts with SARS-CoV-2 S1 and the RBDs (Fig. 5b, Green box). In addition, the vaccine induced antibodies against spike cross reacts with SARS S1 and SARS RBD (Fig. 5b, Blue Box). The complete correlation coefficient matrices can be found in Supplementary Table 3.

As shown here and previous work from our group^6,7^ the specific antibody background reactivity to the novel coronavirus (SARS, MERS, and the SARS-CoV-2) is low in naive populations and rises in response to the infection. However, during natural exposure, cross-reactivity was only observed between SARS-CoV-2 and SARS nucleocapsid proteins or MERS full length spike and SARS-CoV-2 S2 (or full length) was observed. Although it is possible to discover SARS-CoV-2 peptide epitopes that cross-react with peptide epitopes from common CoV^20^, the results in Fig. 5, and Supplementary Table 3, emphasize the low level of cross reactivity against common CoV and flu conformational epitopes represented on the COVAM.

### Nucleocapsid protein is a biomarker associated with natural exposure

Unlike the natural exposure group that reacts uniformly to both nucleocapsid protein and full-length spike, vaccinees can be separated into two distinct groups of those who react to NP and those who do not. Natural exposure induces a dominant antibody response against the nucleocapsid protein (NP), but since NP is not in the vaccine, there is no vaccine induced response against it. In this way, vaccinated people who had a prior natural exposure can be classified because they have antibodies to NP. Vaccinated people who were never previously exposed lack antibodies against NP and vaccinated healthcare workers can be separated into NP negative and NP positive groups.

**Fig. 6:**
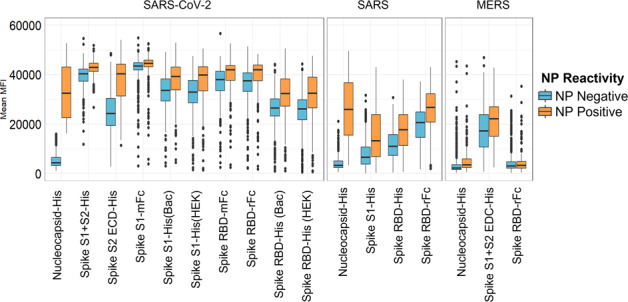
COVAM antibody reactivity of SARS-CoV-2 nucleoprotein seropositive vs seronegative specimens. The boxes represent the first quartile, median and third quartile, and the whiskers extend 1.5 times the interquartile range (IQR). Unlike the natural exposure group that reacts uniformly to both nucleoprotein and full-length spike, vaccinees can be separated into two distinct groups, those who react to NP and those who do not. Natural exposure induces a dominant antibody response against the nucleocapsid protein (NP), but since NP is not in the vaccine, there is no vaccine induced response against it. In this way vaccinated people who had a prior natural exposure can be classified because they have antibodies to NP. Vaccinated people who were never previously exposed lack antibodies against NP. This data further supports the directive that people who are previously exposed will benefit by getting a boost against RBD.

The results in Fig. 6 show the comparison between the antibody responses against the novel coronavirus antigens between the NP positive and NP negative vaccinees. NP reactive individuals show a higher reactivity to the spike antigens, including cross-reactivity against SARS spike, and a lesser degree MERS. This observation further supports advice that people who were previously exposed benefit from getting vaccinated as the antibody response can be further boosted by the vaccine.

### Progression of the prime and boost responses differ between individuals

**Fig. 7:**
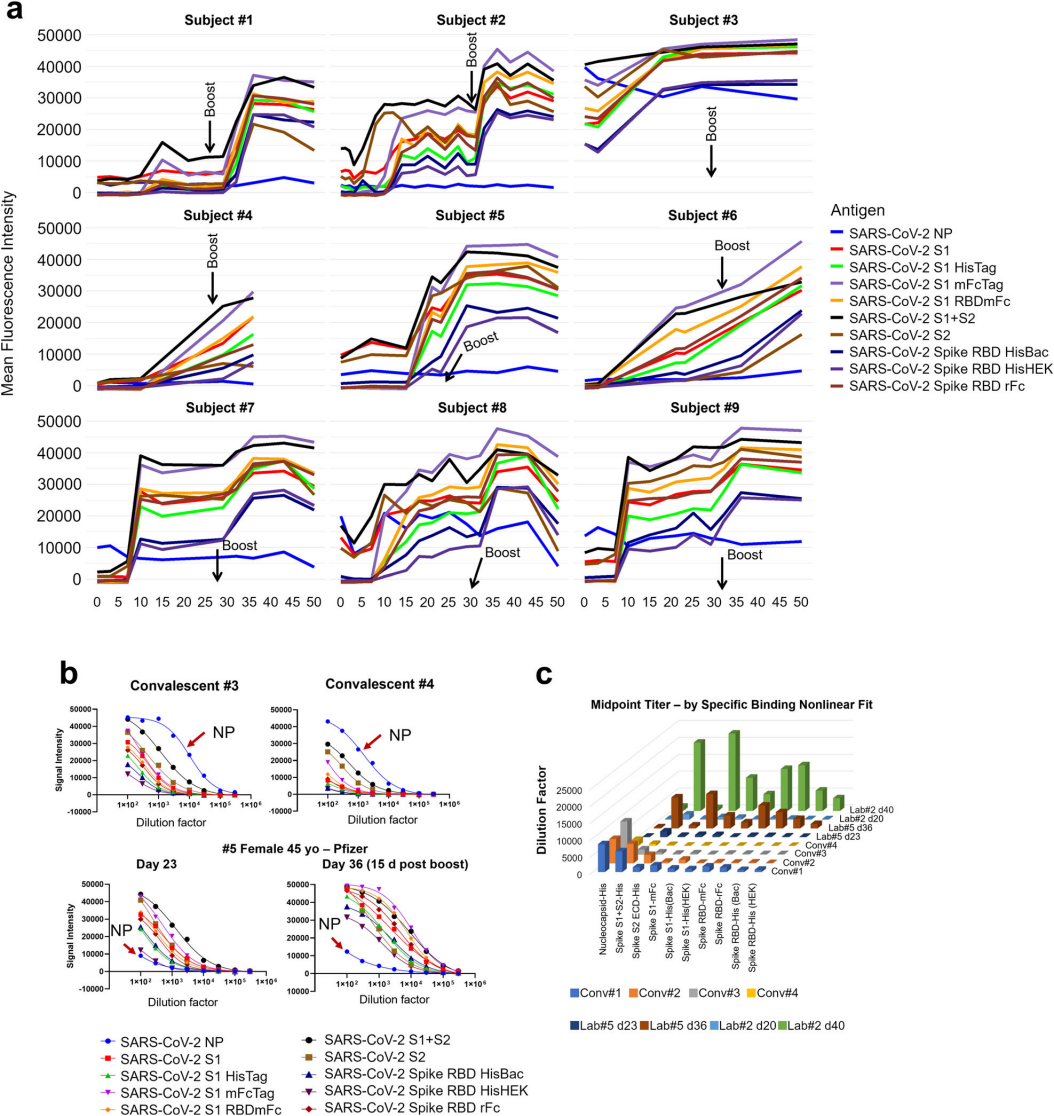
Longitudinal analysis pre and post-mRNA vaccination. **a** Longitudinal specimens taken at weekly intervals from 9 individuals pre- and post-mRNA vaccination. Individuals differ substantially in their response to the prime. Five individuals had low baseline NP reactivity that did not change post-vaccination. Four individuals had elevated NP reactivity at baseline which also did not change significantly post-vaccination; subject #3 was a recovered confirmed COVID case. In this small group, higher baseline NP predicts a higher response after the prime. These results support a directive to get the boost in order to achieve more uniform protection within a population of individuals. **b** Convalescent plasmas from 2 recovered COVID cases, and pre- and post-boost specimens from Subject #5 were titered and the titration curves are shown. The curves are generated by making 8 half log serial dilutions of the plasmas before probing 8 separate COVAM arrays. These curves highlight the observation that high titers against NP are present in convalescent plasma that are lacking in the vaccinees. (red arrow). **c** The midpoint titers of 10 SARS-CoV-2 antigens from 4 convalescent plasmas and plasmas from 2 vaccinees after the prime and after the boost are plotted Convalescent plasmas vary in their titers against NP and full-length spike. The vaccinees lack antibody against NP and have significantly higher titers after the boost against all of the spike antigens compared to convalescent plasma.

Figure 7 shows results of longitudinal specimens taken at varying intervals from 9 individuals pre- and post-mRNA vaccination. Everyone received two doses of the vaccine, a prime and a boost roughly 4 weeks after the primary dose. The data show that the time course of development of the antibody response varies between each individual. There was no significant vaccine induced increase in NP reactivity as expected. The subjects showed either a plateau in the reactivity 5–10 days after the boost dose or a slight decrease in reactivity. It is not yet clear whether this decrease is a sign of the waning antibody response.

Five individuals had low baseline NP reactivity that did not change post-vaccination. Four individuals had elevated NP reactivity at baseline which did not change significantly post-vaccination, and one of these individuals was a confirmed recovered COVID case. Subject #1 had a weak response to the prime and a stronger response to the boost. #2 responded with a strong reactivity to both the prime and the boost with a clear increase in antibody levels for the spike variants. #3 is a recovered confirmed COVID-19 case. As expected, this individual showed an elevated baseline antibody reactivity against NP and all of the SARS-CoV-2 variants. After the first dose, the individual showed an increase in antibody reactivity, however, no further increase was observed after the boost dose. #4 responded slowly to the prime. Subjects #7, #8, and #9 had elevated NP at baseline and responded rapidly to the prime without significant further increase after the boost.

### Anti-spike antibody titers induced by the mRNA vaccine are higher than those induced by natural exposure

COVAM measurements taken at a single dilution of plasma can be used as a parameter to compare relative antibody titers between individual specimens. This is useful for high throughput studies and allows for the probing of thousands of samples in a relatively short time, with minimum staff, and can provide fast and inexpensive data for epidemiology studies to quantify virus exposure levels. However, to obtain a more precise measure of antibody levels, samples can also be titered by serial dilution. In Fig. 7b, 2 convalescent plasmas from recovered COVID cases, and pre- and post-boost vaccination plasmas from Subject #5 were titered. The curves are generated by making 8 half-log serial dilutions of the plasmas before probing the COVAM arrays. These curves highlight the observation that high titers against NP are present in convalescent plasma that are lacking in the vaccinees.

Figure 7c plots the midpoint titers of 10 SARS-CoV-2 antigens in 4 convalescent plasmas and pre- and post-boost plasmas from 2 vaccinees. As expected, convalescent plasmas vary in their titers against both NP and full-length spike. The convalescent plasmas #1 and #2 showed a higher midpoint titer for both NP and full-length spike when compared to the plasmas #3 and 4. Both vaccines showed no antibody reactivity against NP before and after immunization. Although both individuals showed low antibody titer against SARS-CoV-2 antigens right after the primary immunization, both showed significantly higher titers after the boost against all of the spike antigens including S1 and the RBDs, compared to convalescent plasma (Fig. 7c). A summary of the midpoint titers is available in Supplementary Table 1.

## Discussion

In this study, we compared antibody responses induced after SARS-CoV-2 natural exposure with the responses induced by the mRNA vaccines. Pre-vaccine natural exposure data were obtained from two large serial cross-sectional surveys of residents from Orange County and the city of Santa Ana, CA^10^, and from mRNA vaccinated healthcare workers at the UCI Medical Center participating in an intensive vaccination campaign. Within weeks of administration, the mRNA vaccines induced higher antibody levels against spike proteins than observed after natural exposure. These results coincide with equally remarkable clinical trial data showing rapid induction of mRNA protective efficacy on a similar timescale^1,2^. The UCI Medical Center achieved a very rapid introduction of the vaccine beginning on December 16, 2020. Within 5 weeks 78% of the individuals tested were seropositive for spike and 3 months later 99% of a March 2021 cross sectional sample was positive. These results illustrate the high vaccine uptake and the extent of antibody response to the vaccine in this population.

mRNA vaccines induce higher antibody levels and greater antibody breadth than natural exposure to infection and differences were particularly notable against the RBD domain. Out of a collection of 3473 specimens collected from the Santa Ana Cares study in December 2020 we classified 920 as seropositive due to natural exposure before the vaccine was introduced. In February we had a similar number of vaccine induced seropositive healthcare workers. The virus uses the spike RBD domain that binds to the ACE2 receptor on respiratory cells to enter and infect them. Vaccinated individuals had significantly elevated antibody levels against RBD domain segments, supporting the protective immunity induced by this vaccine as previously published^1,2^.

In addition to inducing increased antibody levels against SARS-CoV-2 RBD, the mRNA vaccine induced cross-reactive responses against SARS spike and SARS RBD. Conversely, natural exposure did not induce a cross-reactive response against the SARS spike and SARS RBD. The weak anti-RBD response induced by natural exposure may provide a mechanism for new variants to enter the population. Importantly, the mRNA vaccine induces a marked cross-reactive response against SARS spike, we hypothesize that on possible explanation is that the mRNA vaccine protein adopts a conformation that promotes or facilitates the recognition of the cross-reactive epitopes by the immune system. This effect of the mRNA vaccine to induce cross-reactivity against diverse CoV strains is encouraging, providing further evidence that it may be effective against emerging virus variants. This can be further corroborated by the antibody reactivity against RBD variants from several SRAS-CoV-2 variants. As shown in Fig. 4b, the mRNA vaccinees display a strong antibody response to several RBD variants, here analyzed variants from the lineages B.1.351, B.1.617, B.1.1.7 and Minkvariant (Pango Lineages), all at levels comparable to the Wild Type RBD.

Antibodies induced by natural exposure against the NP from both SARS-CoV-2 and SARS is concordant with an R^2^ value of 0.93. This observation is consistent with the high sequence identity (Around 80%) between these two strains. Conversely, the anti-spike response induced by natural exposure does not cross-react against SARS spike or SARS RBD domain, this observation may indicate different immune selection pressure across these strains because of the importance of this epitope in the infection process.

Anti-nucleocapsid antibody is a biomarker of natural exposure to SARS-CoV-2 and can be used to distinguish individuals in a vaccinated population who have been previously exposed to the virus. The nucleoprotein is not present in currently used vaccines. Our data also suggest that people who have had a prior exposure to the virus mount a stronger immune response to the vaccine than those whose immune response has not yet been primed by a previous exposure or vaccination.

These results also have relevance for both the dose-response hypothesis and regarding herd immunity. Several authors have suggested that disease outcomes may be related to the dose inoculum, with individuals being exposed to inocula with higher virus loads potentially having more severe disease outcomes^21^. While the currently used vaccines in this setting do not rely on viral materials, they do offer a glimpse into controlled high-level exposure to proteins that are specific to SARS-CoV-2. Our results show that individuals who have been vaccinated mount higher across-the-board antibody responses than those who have been exposed to variable viral inocula (i.e., through natural exposure). Second, although more work needs to be done, the variable antibody responses among the pre-vaccine population may also indicate a more variable, and also suggestive of a less intense antibody dependent protection, when compared to vaccinated individuals. This could also indicate that immunity from naturally acquired infections is not as strong as that acquired from vaccination, with potential relevance for reaching and maintaining herd immunity. We should not assume that previously infected individuals are immune or that they cannot transmit the virus.

The original influenza nucleic acid vaccination report published nearly 30 years ago, used the nucleoprotein antigen from influenza because it was conserved across influenza subtypes and it would therefore be a more universal vaccine^12^. This nucleocapsid based nucleic acid vaccine induced cross-subtype protection against both group 1 and group 2 viruses (A/PR/8/34 (H1N1) and A/HK/68 (H3N2)), and it implicated a cell mediated component, killing of infected cells, in the observed efficacy. As reported for influenza, a more universal SARS CoV vaccine may include the nucleocapsid protein antigen.

Individuals differ in the progression of response to the mRNA prime and boost. Some have a weak response to the prime and experience a substantial effect of the boost. To account for these differences, the group of vaccinees that are NP positive also have significantly higher vaccine induced responses than the NP negative individuals. This effect is also evident from the small sample of longitudinal specimens were collected from lab members, those with elevated baseline NP reacted more rapidly against the antigens. In this small sample of longitudinal specimens, anti-spike antibody titers induced by the mRNA vaccine are higher than those induced by natural exposure.

Serological assays for SARS-CoV-2 are of critical importance to identify highly reactive human donors for convalescent plasma therapy, to investigate correlates of protection, and to measure vaccine efficacy and durability. Here we describe results using a multiplex solid phase immunofluorescent assay for quantification of human antibodies against 37 antigens from SARS-CoV-2, other novel and common coronaviruses, and influenza viruses that are causes of respiratory infections. This assay uses a small volume of blood derived from a finger stick, does not require the handling of infectious virus, quantifies the level of different antibody types in serum and plasma and is amenable to scaling. Finger stick blood collection enables large scale epidemiology studies to define the risk of exposure to SARS-CoV-2 in different settings. Since the assay requires 1 microliter of blood it is also practical for monitoring immunogenicity in small animal models. After probing more than 8000 pre- and post-vaccination specimens our results confirm that the mRNA vaccine can be used in an intensive comprehensive vaccination campaign to rapidly immunize large groups.

The concept of nucleic acid vaccines appeared 30 years ago after it was shown that plasmid DNA and RNA could be injected into mouse skeletal muscle tissue in vivo and the encoded transgenes were expressed at the injection site^22,23^. DNA vaccines were the first nucleic acid vaccines to be manufactured and tested on a pharmaceutical scale^17,18^. The mRNA vaccines that are being distributed so widely today may seem to have suddenly emerged, but there has been 30 years of scientific discovery, discourse, and development, work from hundreds of scientists, numerous biotechnology companies, and billions of public and private dollars invested enabling this effective response with a vaccine at this moment.

## Methods

### COVID seroprevalence surveys in Orange County, California

Here we analyzed data from ongoing serologic surveys of healthcare workers (HCW) from the University of California Irvine Medical Center (UCIMC, Orange County, CA, USA) and from residents of the Orange County community. The first community survey (actOC) conducted in July of 2020, was county-wide, and recruitment was done via a proprietary phone list. This survey of 2979 individuals was meant to be representative of the age, ethnicity, and socio-economic makeup of the county (detailed in ref. ^10^). The results of this county-wide survey indicated that the city of Santa Ana was a COVID-19 hotspot, especially on the Hispanic population. Surveillance of reported cases and test positivity corroborated this finding. A second, seroprevalence survey was then conducted in Santa Ana as the Santa Ana Cares study in December of 2020. Recruitment of 3347 individuals for this second survey was done using randomized house sampling within census tracts coupled with a community engaged campaign with support from Latino Health Access (a community-based health organization that has been based in Santa Ana for over 2 decades, https://www.latinohealthaccess.org/). Analysis of the second seroprevalence survey is ongoing. While the first survey was county-wide, the serological test positivity reported in this analysis come from zip codes in Santa Ana alone.

Samples were also collected from the UCIMC longitudinal HCW study in May and December 2020. A comprehensive mRNA vaccination campaign started at UCIMC on December 16 2020 and 6,724 HCW were vaccinated in 3 weeks. Three additional cross-sectional samples were taken at end of January, February, and March 2021. A descriptive demographics able for both (Santa Ana and Health Care Workers) cohorts is available in Supplementary Table 4.

An informed consent was obtained from all participants. All methods were performed in accordance with the relevant guidelines and regulations. All methods were approved by the CI Institutional Review Board (HS# 2020-5952 and HS# 2020–5818), and Comprehensive Clinical, Imaging, and Histological Database for the Study of COVID-19 Infection and Outcomes (HS# 2020-5783).

A Coronavirus Antigen Microarray (COVAM) was used to measure antibody levels against 37 antigens from coronaviruses and influenza. COVAM measurements taken at a single dilution of plasma can be used as a parameter to compare relative antibody titers between individual specimens against each of the individual 37 antigens. The COVAM contained 10 SARS-CoV-2, 4 SARS, 3 MERS, 12 Common CoV, and 8 influenza antigens. (Fig. 1) Samples were probed and analyzed on the COVAM and each individual was provided with the results of their test (Supplementary Section) according to the IRB protocol^6^. Samples were diluted in a 1:200 ratio with Blocking buffer, Quantum dot conjugated antibodies (Goat Anti-Human IgG Fc conjugated to Quantum dot 800, GraceBio, SKU 110610; Goat F(ab’)2 Anti-Human IgM conjugated to Quantum dot 655, GraceBio, SKU 110630; Goat anti-human IgA conjugated to Quantum dot 655, GraceBio, SKU 110620) were diluted with blocking buffer at a 1:100 ratio.

### Reporting summary

Further information on research design is available in the Nature Research Reporting Summary linked to this article.

## Supplementary information

Supplementary Information

Reporting summary

## Data Availability

The raw data that support the findings of this study are available from the corresponding author upon reasonable request.

## Code availability

The complete custom R scripts used for data analysis are available from the corresponding author upon reasonable request.
