# Supplementary material for: Distinct SARS-CoV-2 antibody reactivity patterns elicited by natural infection and mRNA vaccination
Source: NPJ Vaccines. 2021 Nov 4;6:132. doi: 10.1038/s41541-021-00396-3 (PMC8568980; doi:10.1038/s41541-021-00396-3)
Supplement: Supplementary file 1 — Supplementary Information [file 41541_2021_396_MOESM1_ESM.pdf]

Supplementary Methods

Coronavirus Antigen Microarray (CoVAM) Report

This document describes the pipeline used to analyze the COVAM array and generate the individual reports.

Step 1: Data pre-processing

The first step of the analysis is importing all data into the R environment. The sample set containing the known negative and known positive controls, here named "Control Set", is loaded separately from the sample set being analyses.

Following this step, to prevent errors when addressing specific columns, or samples, all spaces are removed both from the column names from all data sets imported, as well as from the Unique sample IDs reference from the meta data files.

On the data processing steps, the following are performed:

From the raw data, the signal to noise ratio (SNR) is calculated. The SNR is calculated as the median signal intensity of a given spot divided by the background signal of the vicinity surrounding area. For the quality check purposes, the mean SNR is Calculated only for spots with MFI over 20,000. Samples with a mean SNR below 2 are flagged for further visual inspection or for reprobing.

After calculating the men SNR, the control spots are then assessed. First, for each sample, and each antigen (printed in triplicates), the first and third quartile as well as interquartile range (IQR) are calculated for the control spots. An upper MFI limit of 1.5 times the IQR over the third quartile and a lower limit of 1.5 times the IQR bellow the first quartile are defined. Spots outside this range are removed and replaced with the mean MFI of the remaining replicates of the spot.

Next, a similar approach is applied to flag samples for which the overall control spots distribution is out of range (2*IQR + third Quartile for the upper limit and first quartile – 2*IQR for the lower limit). For this, all controls spots of a given sample are used. Out of range samples are flagged for further visual inspection or reprobing.

Finally, the printing buffer background reactivity is subtracted from each spot and the samples are normalized.

Step 2: Normalization

Data normalization is performed in two steps. First The control spots are normalized against the training set using the Quantile Normalization method. This allows to calculate a normalization factor that will be used to rescale the data to match the training set and preserving the individual reactivity diversity. After normalizing the control spots, their sum is calculated. A rescaling factor is calculated by dividing the sum of the normalized control spots of the training set by the sum of the normalized control spots of each sample. The resulting factor is then multiplied by the reactivity of each spot resulting in a rescaled data frame. The mean reactivity of the normalized data is then calculated.

Step 3 a: Prediction models

Previous to the sample analysis, the prediction models were constructed using a sample set composed by samples with known diagnosis for COVID-19. These samples are both Negative controls (samples collected before the pandemic) and Positive controls (Samples from individuals diagnosed for COVID-19 by PCR). This control set is heer referred to as Training Set.

The Construction of the prediction models was performed as following.

1. Data is pre-processed and normalized as described above.
2. The reference data set was decomposed into a vector using the function ‘unmatrix’ from the package gData (version 2.18.0).
3. A mixture model is calculated for the vector using the function ‘normalmixEM’ from the package ‘mixtools’ (version 1.2.0).
4. A cutoff is then calculated as 3 standard deviations over the mean of the negative signal curve.
5. Wilcox test for each antigen was performed comparing the positive controls and negatives control, considering significant, antigens with $p < 0.05$.

following the selection of seropositive antigens, an optimal predictive combination of these antigens was selected. (that left us with 7 antigens as seropositive for IgG, and 8 for IgM).

The selection was performed as follows:

1. For every possible combination of the seropositive SARS-CoV-2 antigens from 1 all (7 for IgG and 8 for IgM), the reference set was randomly divided into a training and a testing sets at a 70%/30% ratio.
2. A logistic regression was generated using the reference set. The regression was generated using the function ‘glm’ of the ‘stats’ package (version 4.0.0).and a ROC curve was calculated (package pROC version 1.16.2).

3. The optimal coordinates of the ROC curve were obtained based on the ‘youden index’, by prioritizing the specificity.
4. The coordinates were obtainded using the function ‘coords’ from the pROC library. The coordinates are obtainded in a table format with each row containing a regression threshold and its related specificity and sensitivity.
5. The coordinates were then subset to represent specificities of 0.95 or higher. A threshold was then defined as the threshold on the coordinate with the highest specificity on the subset.
6. A logistic regression was then calculated using the testing set and each sample classified as negative or positive by comparison with the threshold.
7. A confusion matrix was calculated by comparing the predicted outcomes and the known classifications (“known negative” or “Known positive”) and the prediction specificity and sensitivity stored into a vector.
8. This analysis was repeated 1000 times and the sensitivity and sensitivity calculated as the mean predicted performance of all repetitions.

The performance outcome for each antigen combination was analyzed and a selection of the best performing combinations was made based on the specificity and sensitivity. The selected candidates were then tested using the full reference sample set. The test was performed as follows:

1. A logistic regression for each antigen combination candidate using the full reference set. Then a ROC curve was calculated and the coordinate table with all curve points was obtained.
2. The coordinates of each candidate were compared in order to select the candidate with the highest sensitivity, given a fixed specificity of 1 (100%).

In addition to the logistic regression model, a Random Forest model was constructed using all reactive antigens.

Step 3 b: Reports.

After Data Normalization, the predictions models, constructed as described above, are loaded and reactivity predictions are performed using Random Forest and Logistic Regression for the multi antigen combinations. In addition to the multi antigen predictions, a prediction for each single SARS-CoV-2 antigen was performed for every sample, for both IgG, and IgM. These predictions were performed using the threshold calculated using the optimal 'youden' index. Every sample can be classified as reactive or not reactive for each single SARS-CoV-2 antigen.

The report phase consists on the output of single pdf files with the individual subject predictions and interpretation. The file consists on a brief explanation of the array on the first page, as well as some information on the performance of the array with the current settings. In addition, on the first page there is a short disclaimer of the scope and limitations of the assay.

The second page consists of a table for all the SARS-CoV-2 antigens with their ROC predictions. These predictions are for a qualitative understanding of one's reactivity and may not directly correlate with the multi antigen prediction.

The Multi antigen prediction, or the sample classification into the three reactive groups, is presented also on a short table displaying the prediction of IgG and IgM separately. The overall sero-reactivity of the sample to all antigens is depicted on two graphs on the second page. One showing the reactivity for IgG and one for IgM.

On each graph, the individual`s reactivity is represented as dots with its standard errors. For reference, a red line representing the positive control mean reactivity with its confidence interval, as well as a blue line representing the negative controls mean reactivity with its confidence interval are also plotted.

Supplementary Figure 1

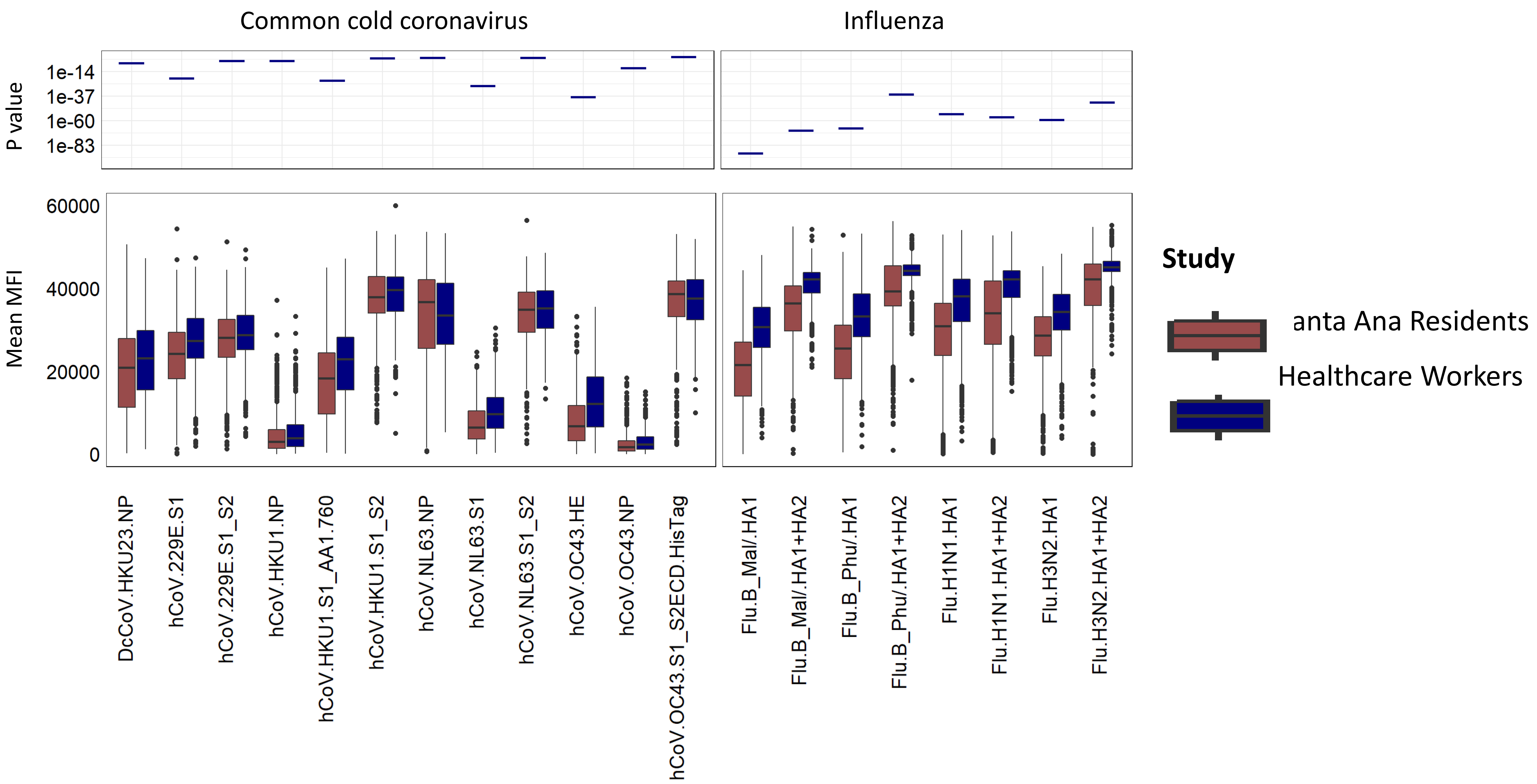

Supplementary Figure 1. Natural Exposure vs mRNA Vaccination antibody Reactivity against Common Cold Coronavirus and Influenza antigens.

Mean MFI signals for the common coronaviruses and Influenza antigens in the natural exposure cohort from Santa Ana in December 2020 (actOC) and the February 2021 vaccination group (HCW) are plotted. The boxes represent the first quartile, median and third quartile and the whiskers extend 1.5 times the interquartile range (IQR). Wilcoxon test was performed for pairwise comparisons. The figure shows that antibody responses against common cold antigens are not significantly different in both populations. A relatively higher reactivity was for the UCIMC group was observed for the influenza antigens.

Supplementary Figure 2
Analysis method and report generation

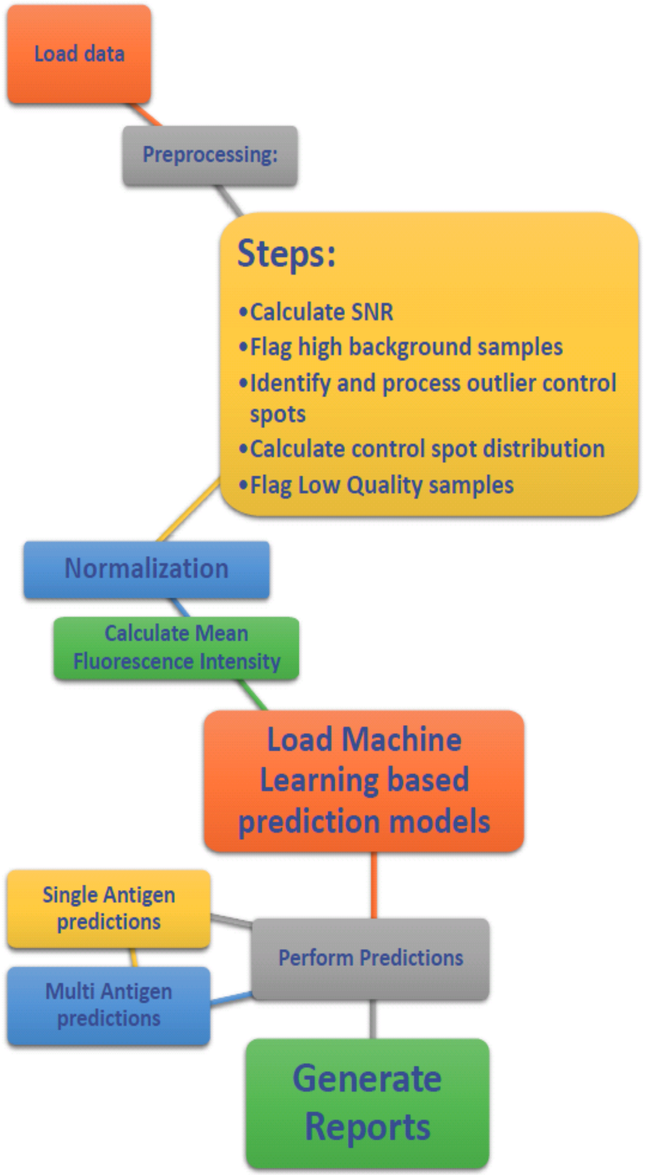

Supplementary Figure 2. General COVAM analysis pipeline

The general analysis pipeline consists of three main steps: the preprocessing, the normalization and then the statistical prediction analysis. The preprocessing includes steps like calculation the Signal to Noise Ratio (SNR) and determine if a sample needs to be further checked or re-assayed (due to the background reactivity levels). If successful, samples are successful analyzed for their SNR, the controls spots are checked to remove outlier spots that could skew normalization. Then, the distribution of the control spots is analyzed and low-quality samples (for which the control spots deviate from the expected) are flagged to be re-assayed. Then the samples are normalized, and the mean fluorescence intensity calculated from the average of the 3 replicates in the array. After normalization, a machine learning based algorithm is used to classify each sample as reactive or not reactive to SARS-CoV-2 (using multiple antigens) as well as to individual antigens. Then, individual reports are generated for each sample (this can be in the form of individual pdf files that may be delivered to the subject).

Supplementary Figure 3

10,000 Individual Patient Specific Reports

Subject: 754551

Single Antigen Logistic Regression

Antigen	IgG	IgM
SARS.CoV.2.NP	Not Reactive	Not Reactive
SARS.CoV.2.S1	Not Reactive	Not Reactive
SARS.CoV.2.S1.HisTag	Not Reactive	Not Reactive
SARS.CoV.2.S1.mFcTag	Not Reactive	Not Reactive
SARS.CoV.2.S1.RBD.mFc	Not Reactive	Not Reactive
SARS.CoV.2.S1+S2	Not Reactive	Not Reactive
SARS.CoV.2.S2	Not Reactive	Not Reactive
SARS.CoV.2.Spike.RBD.His.Bac	Not Reactive	Not Reactive
SARS.CoV.2.Spike.RBD.His.HEK	Not Reactive	Not Reactive
SARS.CoV.2.Spike.RBD.rFc	Not Reactive	Not Reactive

SARS−CoV−2 reactivity profile Classification:
(see detailed explanation on previous page)

Multi Antigen prediction

	IgG	IgM
Logistic Reg.	Non−Reactive	Non−Reactive
RandomForest (RF)	Non−Reactive	Non−Reactive
*RF Probability **	26%	15.2%

*Random Forest algorithm probability of classification as 'Reactive'
the probability cutoff is currently set at 70%(IgG)/85%(IgM)

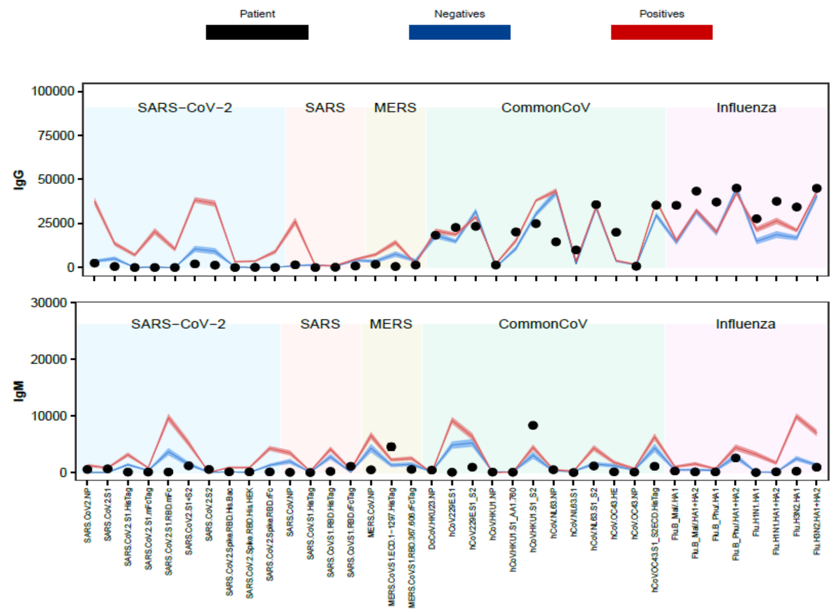

Subject: 755334

Single Antigen Logistic Regression

Antigen	IgG	IgM
SARS.CoV.2.NP	Not Reactive	Reactive
SARS.CoV.2.S1	Reactive	Reactive
SARS.CoV.2.S1.HisTag	Reactive	Not Reactive
SARS.CoV.2.S1.mFcTag	Reactive	Reactive
SARS.CoV.2.S1.RBD.mFc	Reactive	Not Reactive
SARS.CoV.2.S1+S2	Reactive	Reactive
SARS.CoV.2.S2	Not Reactive	Reactive
SARS.CoV.2.Spike.RBD.His.Bac	Reactive	Reactive
SARS.CoV.2.Spike.RBD.His.HEK	Reactive	Not Reactive
SARS.CoV.2.Spike.RBD.rFc	Reactive	Reactive

SARS−CoV−2 reactivity profile Classification:
(see detailed explanation on previous page)

Multi Antigen prediction

	IgG	IgM
Logistic Reg.	Reactive	Reactive
RandomForest (RF)	Reactive	Reactive
*RF Probability **	77.8%	96.2%

*Random Forest algorithm probability of classification as 'Reactive'
the probability cutoff is currently set at 70%(IgG)/85%(IgM)

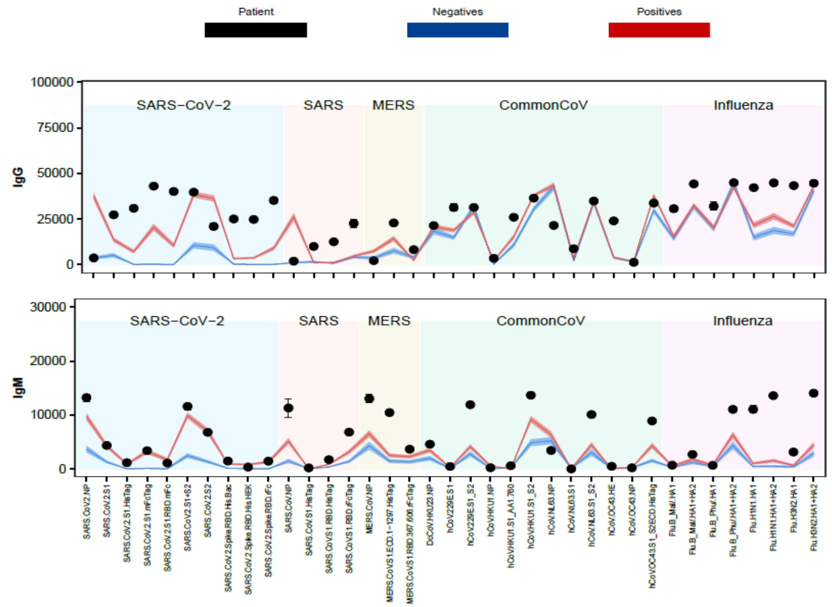

Supplementary Figure 3. Individualized pdf report models.

After the machine learning classification of each sample individual pdf files containing the results can be generated. The panels in the figure are representative of a typical negative (or non-reactive) result (left panel) and of a typical positive (Reactive) sample (on the right). The data printed on the reports are basic reactivity classification for the SARS-CoV-2 antigens (Only reactive and Non-reactive denominations are given). As well as the machine learning classification (multi antigen classification) denominations. For the multi antigen classification, the results from the logistic regression as well as the results from random forest, as well as the random forest probabilities are given. The multi antigen classification is the main result and is the one used to classify an individual as exposed, or reactive to SARS-CoV-2 as individual antigens alone have a much lower performance in the classification. Finally, since the COVAM is composed of multiple viruses, the reactivity to the entire array is given to both IgG and IgM. This reactivity is given as the normalized mean florescence intensities and as a reference, the confidence intervals of a known control set of samples (known positives red line and red bands and known negatives blue line and blue bands) are given. Although these reports give a much more comprehensive view of an individual`s reactivity status to SARS-CoV-2, they are intended mainly as a guidance as the COVAM array is not approved by the FDA as a diagnostic test.

Supplementary Figure 4

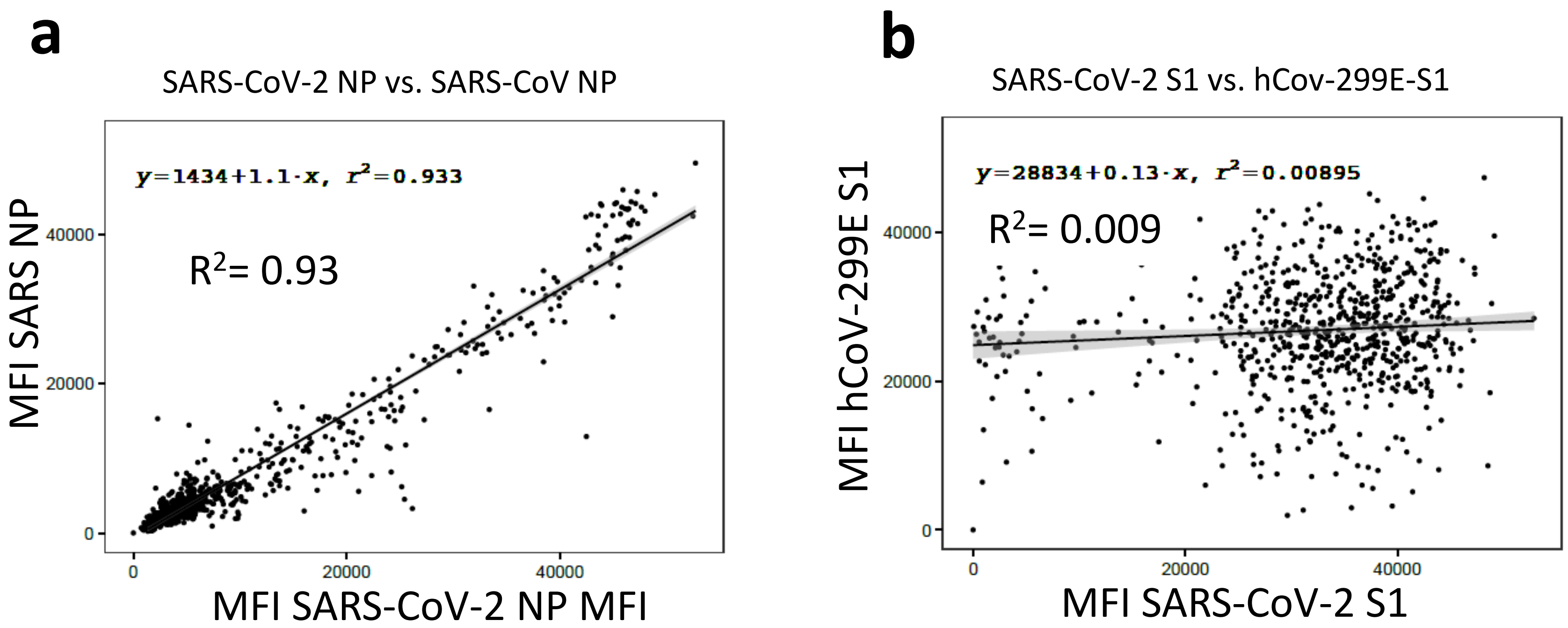

Supplementary Figure 4. Antibody correlation scatterplots.

Scatterplots can be used to compare antibody reactivities of any 2 antigens on the COVAM array. **(A)** There are 920 seropositive specimens from Orange County residents. antibody reactivity against SARS-CoV-2 and SARS NP in this population are well correlated (R^2= 0.93). antibodies against NP from SARS-CoV-2 cross reactive against the NP from SARS. **(B)** antibody reactivities between SARS-CoV-2 S1 and hCoV-299E S1 are not correlated (R^2= 0.009) so antibodies against SARS-CoV-2 S1 do not cross-react against S1 from hCoV-299E. The R^2 value can be used as a metric to determine cross-reactivity between any 2 antigens.

Supplementary Figure 5

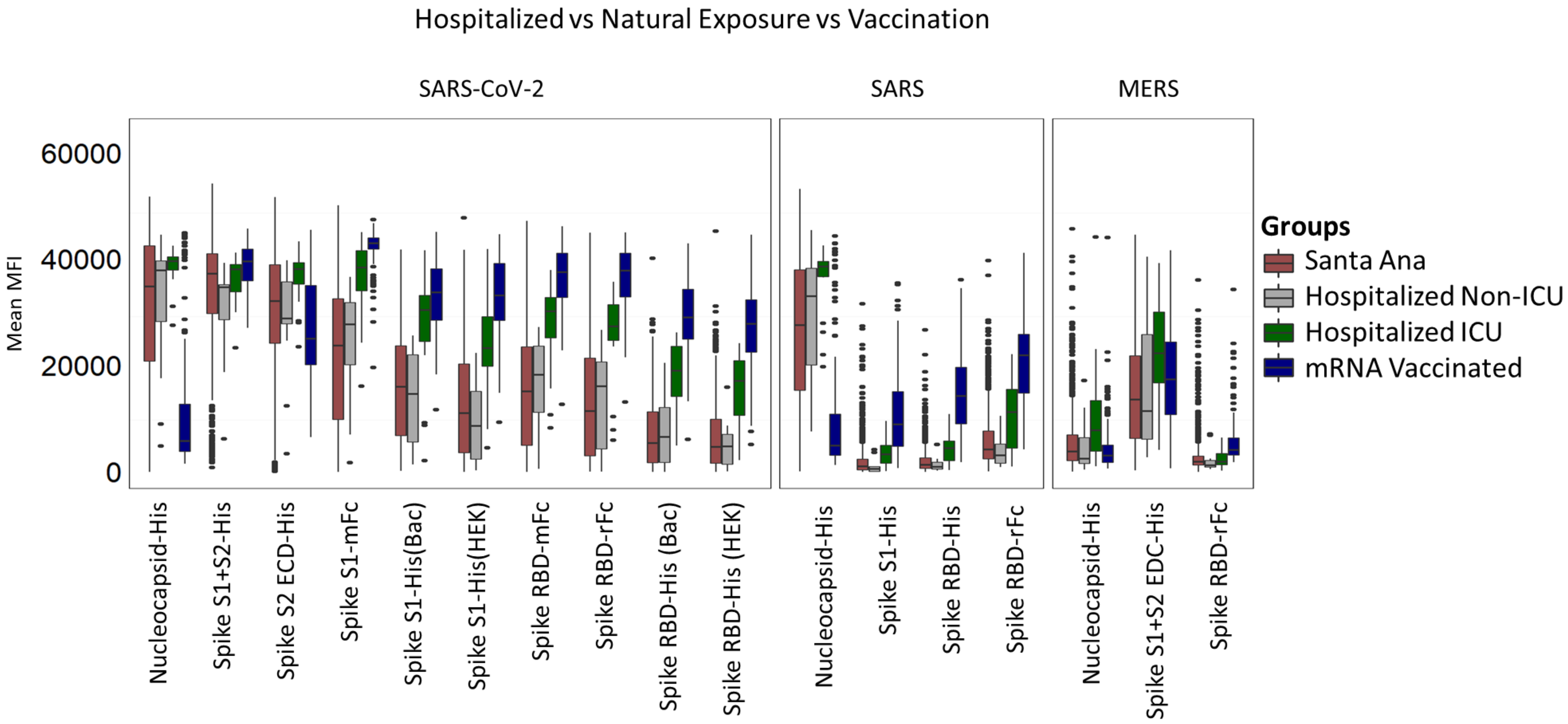

Supplementary Figure 5. Disease Severity vs mRNA Vaccination antibody Reactivity.

Mean MFI signals for each of the novel coronavirus antigens in the natural exposure cohort from Santa Ana in December 2020 (actOC), the February/March 2021 vaccination group (HCW) and samples from hospitalized individuals (stratified into patients that needed Intensive care – ICU- and patients with less severe disease – Non-ICU) are plotted. The boxes represent the first quartile, median and third quartile and the whiskers extend 1.5 times the interquartile range (IQR). The figure shows that antibody levels against Spike S1-RBD from Non-ICU COVID-19 patients are similar to the Santa Ana population while severe COVID-19 patients that needed ICU, had antibody higher antibody levels when compared to non-ICU, although still lower than vaccinated individuals. Vaccination induces a broader and higher titer antibody response than natural exposure alone, so those who have recovered from COVID can be expected to benefit from the vaccination

Supplementary Table 1

Midpoint titer – Specific Binding Saturation Nonlinear Fit

(Dilution Factor)

	Conv#1	Conv#2	Conv#3	Conv#4	Lab#5 d23	Lab#5 d36	Lab#2 d20	Lab#2 d40
**Nucleocapsid-His**	8251	7210	9814	1763	158	328	266	1285
**Spike S1+S2-His**	6161	5790	1549	588	1695	9183	1542	20239
**Spike S2 ECD-His**	1411	2480	631	267	399	884	333	871
**Spike S1-mFc**	1971	468	276	59	668	10166	760	22910
**Spike S1-His(Bac)**	1036	1097	366	151	635	3855	576	9843
**Spike S1-His(HEK)**	835	150	146	53	153	1807	270	4950
**Spike RBD-mFc**	1645	278	272	52	376	6789	384	12442
**Spike RBD-rFc**	1341	213	232	54	346	4815	302	13543
**Spike RBD-His(Bac)**	600	234	139	19	191	2801	171	6039
**Spike RBD-His(HEK)**	692	71	116	3	85	1323	136	3757

Supplementary Table 2

Wilcoxon test p-values

Antigen	Santa Ana vs Hospitalized Non-ICU	Santa Ana vs Hospitalized ICU	Santa Ana vs mRNA Vaccinated	Hospitalized Non-ICU vs Hospitalized ICU	Hospitalized Non-ICU vs mRNA Vaccinated	Hospitalized ICU vs mRNA Vaccinated	HCW vs Santa Ana	HCW NP-Pos vs HCW NP-Neg
SARS.CoV.2.NP	1	1	3.70E-30	1	0.003	8.60E-06	5.28E-146	1.05E-101
SARS.CoV.2.S1+S2	1	1	0.00024	1	0.00091	1	4.44E-11	2.37E-24
SARS.CoV.2.S2	1	1	0.0059	0.7	1	0.026	1.85E-29	2.64E-47
SARS.CoV.2.S1.mFcTag	1	0.0052	1.20E-67	0.027	9.80E-08	0.0032	1.08E-187	2.08E-08
SARS.CoV.2.S1	1	0.0031	2.70E-63	0.038	1.20E-07	1	1.01E-184	7.06E-17
SARS.CoV.2.S1.HisTag	1	0.018	1.60E-69	0.032	6.40E-08	0.0012	6.82E-200	1.73E-18
SARS.CoV.2.S1.RBD.mFc	1	0.0066	2.20E-72	0.12	7.50E-08	0.00015	1.37E-210	1.06E-13
SARS.CoV.2.Spike.RBD.rFc	1	0.0048	7.80E-77	0.12	6.00E-08	7.10E-06	4.95E-221	1.03E-17
SARS.CoV.2.Spike.RBD.His.Bac	1	0.0014	1.20E-79	0.18	6.60E-08	4.40E-06	4.62E-223	3.63E-20
SARS.CoV.2.Spike.RBD.His.HEK	1	0.0096	9.80E-76	0.12	6.20E-08	8.80E-06	4.21E-219	1.30E-19
SARS.CoV.NP	1	0.48	2.50E-27	1	0.0069	8.10E-07	7.85E-147	1.20E-94
SARS.CoV.S1.HisTag	1	0.53	4.80E-61	1	4.40E-07	0.00032	2.90E-168	2.69E-17
SARS.CoV.S1.RBD.HisTag	1	0.041	3.40E-77	0.2	5.20E-08	4.00E-07	8.91E-217	4.19E-18
SARS.CoV.S1.RBD.rFcTag	1	0.45	9.70E-67	0.3	8.90E-08	2.00E-04	1.21E-188	8.77E-18
MERS.CoV.NP	1	1	1	1	1	0.2	5.71E-23	3.01E-15
MERS.CoV.S1.ECD.1-1297.HisTag	1	0.25	0.0053	1	1	1	1.47E-08	4.27E-07
MERS.CoV.S1.RBD.367.606.rFcTag	1	1	3.10E-36	1	0.00016	0.0055	1.78E-36	0.593817
DcCoV.HKU23.NP	1	1	0.044	1	1	1	6.78E-07	0.077335
hCoV.229E.S1	1	1	2.10E-15	1	0.52	0.6	6.03E-21	0.042409
hCoV.229E.S1_S2	1	1	0.12	1	1	1	0.000108	0.013196
hCoV.HKU1.NP	1	1	0.0048	1	1	1	7.80E-05	0.704644
hCoV.HKU1.S1_AA1.760	0.98	1	2.10E-11	1	1	1	5.94E-23	0.754569
hCoV.HKU1.S1_S2	1	1	1	1	1	1	0.051882	2.26E-19
hCoV.NL63.NP	1	1	1	1	1	1	0.065242	2.48E-08
hCoV.NL63.S1	1	1	2.00E-21	1	0.0034	0.00017	3.52E-28	0.880911
hCoV.NL63.S1_S2	1	1	1	1	0.77	1	0.055324	0.075641
hCoV.OC43.HE	1	1	5.10E-30	1	0.0044	0.0053	9.75E-39	0.493176
hCoV.OC43.NP	1	1	5.80E-06	1	1	1	1.94E-11	0.142538
hCoV.OC43.S1_S2ECD.HisTag	1	1	1	1	1	1	0.392556	7.73E-18
Flu.B_Mal/.HA1	1	1	6.00E-39	1	5.00E-05	5.40E-06	2.74E-91	0.202547
Flu.B_Mal/.HA1+HA2	1	1	1.10E-24	1	0.0051	1.00E-05	5.58E-70	0.461181
Flu.B_Phu/.HA1	1	1	9.00E-28	1	2.40E-05	1.40E-06	1.10E-67	0.279288
Flu.B_Phu/.HA1+HA2	1	1	9.80E-11	1	0.00053	1.10E-06	5.76E-36	0.839317
Flu.H1N1.HA1	1	1	3.20E-21	1	0.004	0.18	2.61E-54	0.20701
Flu.H1N1.HA1+HA2	1	1	9.40E-24	1	0.0031	0.17	1.75E-57	0.078836
Flu.H3N2.HA1	1	1	5.40E-15	1	0.0023	0.0019	8.67E-60	0.928062
Flu.H3N2.HA1+HA2	1	1	4.40E-10	1	4.60E-06	2.90E-07	8.98E-44	0.059018

Supplementary table 3 – Santa Ana, Orange County

December 2020, Orange County, Santa Ana

	SARS.CoV.2.NP	SARS.CoV.2.S1+S2	SARS.CoV.2.S2	SARS.CoV.2.S1.mFcTag	SARS.CoV.2.S1	SARS.CoV.2.S1.HisTag	SARS.CoV.2.S1.RBD.mFc	SARS.CoV.2.Spike.RBD.rFc	SARS.CoV.2.Spike.RBD.His.Bac	SARS.CoV.2.Spike.RBD.His.HEK	SARS.CoV.NP	SARS.CoV.S1.HisTag	SARS.CoV.S1.RBD.HisTag	SARS.CoV.S1.RBD.rFcTag	MERS.CoV.NP	MERS.CoV.S1.ECD.1-1297.HisTag	MERS.CoV.S1.RBD.367.606.rFc	DcCoV.HKU23.NP	hCoV.229E.S1	hCoV.229E.S1_S2	hCoV.HKU1.NP	hCoV.HKU1.S1_AA1.760	hCoV.HKU1.S1_S2	hCoV.NL63.NP	hCoV.NL63.S1	hCoV.NL63.S1_S2	hCoV.OC43.HE	hCoV.OC43.NP	hCoV.OC43.S1_S2ECD.HisTag	Flu.B_Mal/.HA1	Flu.B_Mal/.HA1+HA2	Flu.B_Phu/.HA1	Flu.B_Phu/.HA1+HA2	Flu.H1N1.HA1	Flu.H1N1.HA1+HA2	Flu.H3N2.HA1	Flu.H3N2.HA1+HA2
SARS.CoV.2.NP	1																																				
SARS.CoV.2.S1+S2	0.366	1																																			
SARS.CoV.2.S2	0.379	0.951	1																																		
SARS.CoV.2.S1.mFcTag	0.445	0.517	0.556	1																																	
SARS.CoV.2.S1	0.133	0.629	0.626	0.305	1																																
SARS.CoV.2.S1.HisTag	0.327	0.411	0.449	0.916	0.295	1																															
SARS.CoV.2.S1.RBD.mFc	0.406	0.467	0.503	0.955	0.29	0.929	1																														
SARS.CoV.2.Spike.RBD.rFc	0.362	0.425	0.457	0.91	0.281	0.944	0.959	1																													
SARS.CoV.2.Spike.RBD.His.Bac	0.321	0.485	0.513	0.797	0.46	0.882	0.851	0.866	1																												
SARS.CoV.2.Spike.RBD.His.HEK	0.298	0.351	0.381	0.828	0.268	0.947	0.897	0.929	0.908	1																											
SARS.CoV.NP	0.901	0.407	0.429	0.495	0.143	0.342	0.446	0.389	0.341	0.321	1																										
SARS.CoV.S1.HisTag	0.001	0.204	0.218	5E-04	0.577	3E-04	3E-04	2E-04	0.053	1E-04	0.001	1																									
SARS.CoV.S1.RBD.HisTag	0.037	0.337	0.342	0.048	0.704	0.047	0.047	0.046	0.18	0.04	0.032	0.8	1																								
SARS.CoV.S1.RBD.rFcTag	0.02	0.303	0.3	0.03	0.649	0.026	0.029	0.042	0.13	0.023	0.018	0.694	0.846	1																							
MERS.CoV.NP	0.028	0.044	0.048	0.02	0.065	0.02	0.021	0.02	0.029	0.016	0.036	0.055	0.06	0.06	1																						
MERS.CoV.S1.ECD.1-1297.HisTag	0.076	0.568	0.579	0.104	0.696	0.086	0.095	0.085	0.162	0.065	0.079	0.542	0.601	0.583	0.071	1																					
MERS.CoV.S1.RBD.367.606.rFcTag	2E-04	0.176	0.168	0.001	0.559	0.002	0.002	#####	0.026	0.001	3E-04	0.785	0.72	0.795	0.041	0.55	1																				
DcCoV.HKU23.NP	0.01	0.012	0.01	0.002	0.008	6E-04	4E-04	3E-04	8E-04	1E-04	0.004	0.011	0.023	0.026	0.045	0.047	0.013	1																			
hCoV.229E.S1	0.005	0.013	0.007	8E-04	0.009	0.003	0.004	0.005	0.008	0.006	0.002	0.001	0.009	0.009	0.006	0.017	0.006	0.118	1																		
hCoV.229E.S1_S2	0.01	0.068	0.053	0.001	0.093	0.002	0.004	0.004	0.02	0.005	0.006	0.083	0.113	0.117	0.028	0.132	0.107	0.147	0.824	1																	
hCoV.HKU1.NP	0.005	0.009	0.007	#####	0.008	4E-04	2E-04	5E-04	0.005	8E-04	#####	0.008	0.025	0.028	0.059	0.031	0.014	0.483	0.079	0.094	1																
hCoV.HKU1.S1_AA1.760	0.039	0.007	0.005	4E-04	0.001	5E-04	0.002	9E-04	0.001	4E-04	0.022	#####	0.007	0.003	0.005	0.01	#####	0.112	0.176	0.159	0.173	1															
hCoV.HKU1.S1_S2	0.094	0.216	0.183	0.052	0.173	0.044	0.049	0.051	0.081	0.038	0.08	0.072	0.156	0.18	0.027	0.209	0.081	0.131	0.14	0.255	0.106	0.251	1														
hCoV.NL63.NP	0.046	0.029	0.02	0.002	0.018	4E-04	0.002	0.001	0.005	0.002	0.037	0.008	0.024	0.022	0.04	0.037	0.013	0.186	0.154	0.215	0.105	0.1	0.216	1													
hCoV.NL63.S1	0.03	0.029	0.023	0.006	0.02	0.011	0.014	0.019	0.017	0.02	0.019	0.006	0.018	0.014	0.006	0.049	0.014	0.082	0.242	0.198	0.096	0.166	0.128	0.106	1												
hCoV.NL63.S1_S2	0.022	0.054	0.044	0.001	0.055	0.002	0.003	0.005	0.011	0.003	0.014	0.045	0.064	0.08	0.012	0.117	0.071	0.126	0.284	0.387	0.072	0.168	0.368	0.245	0.552	1											
hCoV.OC43.HE	0.014	0.022	0.016	0.002	0.014	0.005	0.006	0.008	0.009	0.008	0.005	0.006	0.018	0.012	0.001	0.03	0.011	0.129	0.134	0.118	0.091	0.138	0.104	0.055	0.158	0.135	1										
hCoV.OC43.NP	0.006	0.01	0.005	0.003	0.005	0.002	0.001	8E-04	#####	7E-04	8E-04	0.011	0.025	0.017	0.032	0.031	0.014	0.581	0.088	0.084	0.507	0.074	0.048	0.086	0.126	0.065	0.177	1									
hCoV.OC43.S1_S2ECD.HisTag	0.09	0.223	0.191	0.044	0.133	0.032	0.042	0.04	0.054	0.027	0.085	0.056	0.111	0.111	0.034	0.221	0.065	0.279	0.173	0.278	0.144	0.215	0.519	0.18	0.189	0.37	0.227	0.17	1								
Flu.B_Mal/.HA1	0.006	0.014	0.006	5E-04	0.016	8E-04	0.001	0.002	0.004	8E-04	0.004	0.012	0.018	0.021	0.026	0.03	0.02	0.133	0.116	0.158	0.1	0.124	0.178	0.111	0.151	0.228	0.077	0.083	0.208	1							
Flu.B_Mal/.HA1+HA2	0.012	0.035	0.022	0.004	0.029	0.004	0.006	0.007	0.011	0.003	0.011	0.012	0.025	0.044	0.015	0.053	0.026	0.123	0.168	0.28	0.058	0.135	0.34	0.191	0.137	0.438	0.083	0.027	0.328	0.644	1						
Flu.B_Phu/.HA1	0.003	0.027	0.017	#####	0.021	2E-04	4E-04	0.001	0.004	4E-04	0.003	0.022	0.034	0.048	0.026	0.057	0.041	0.131	0.112	0.2	0.106	0.08	0.204	0.1	0.115	0.256	0.106	0.062	0.248	0.517	0.501	1					
Flu.B_Phu/.HA1+HA2	3E-04	0.006	0.004	3E-04	0.006	5E-04	3E-04	3E-04	8E-04	1E-04	0.001	8E-04	0.002	0.011	0.011	0.014	0.006	0.056	0.065	0.164	0.011	0.037	0.261	0.118	0.034	0.295	0.027	7E-04	0.176	0.22	0.548	0.36	1				
Flu.H1N1.HA1	5E-04	0.014	0.007	3E-04	0.013	5E-04	#####	#####	#####	5E-04	5E-04	0.008	0.017	0.038	0.022	0.049	0.021	0.087	0.099	0.183	0.052	0.067	0.183	0.13	0.087	0.25	0.034	0.024	0.192	0.223	0.378	0.268	0.253	1			
Flu.H1N1.HA1+HA2	3E-04	0.012	0.007	#####	0.01	#####	3E-04	#####	9E-04	#####	0.001	0.004	0.012	0.03	0.023	0.032	0.013	0.077	0.092	0.177	0.042	0.041	0.154	0.114	0.041	0.198	0.015	0.013	0.148	0.162	0.361	0.262	0.375	0.761	1		
Flu.H3N2.HA1	0.005	0.01	0.006	5E-04	0.011	0.003	0.002	0.005	0.005	0.004	0.002	0.003	0.014	0.019	0.003	0.028	0.011	0.105	0.115	0.166	0.095	0.069	0.217	0.139	0.104	0.251	0.082	0.06	0.211	0.231	0.331	0.308	0.237	0.31	0.23	1	
Flu.H3N2.HA1+HA2	0.005	0.007	0.003	#####	0.003	#####	1E-04	2E-04	7E-04	#####	0.003	5E-04	0.005	0.01	0.005	0.011	0.005	0.086	0.093	0.2	0.023	0.046	0.227	0.189	0.029	0.285	0.03	0.014	0.204	0.152	0.433	0.266	0.672	0.304	0.441	0.405	1

Supplementary table 3 – Health Care Workers

February 2021, Orange County, Santa Ana

	SARS.CoV.2.NP	SARS.CoV.2.S1+S2	SARS.CoV.2.S2	SARS.CoV.2.S1.mFcTag	SARS.CoV.2.S1	SARS.CoV.2.S1.HisTag	SARS.CoV.2.S1.RBD.mFc	SARS.CoV.2.Spike.RBD.rFc	SARS.CoV.2.Spike.RBD.His.Bac	SARS.CoV.2.Spike.RBD.His.HK	SARS.CoV.NP	SARS.CoV.S1.HisTag	SARS.CoV.S1.RBD.HisTag	SARS.CoV.S1.RBD.rFcTag	MERS.CoV.NP	MERS.CoV.S1.ECD.1-1297.HisTag	MERS.CoV.S1.RBD.367.606.rFc Tag	DcCoV.HKU23.NP	hCoV.229E.S1	hCoV.229E.S1_S2	hCoV.HKU1.NP	hCoV.HKU1.S1_AA1.760	hCoV.HKU1.S1_S2	hCoV.NL63.NP	hCoV.NL63.S1	hCoV.NL63.S1_S2	hCoV.OC43.HE	hCoV.OC43.NP	hCoV.OC43.S1_S2ECD.HisTag	Flu.B_Mal/.HA1	Flu.B_Mal/.HA1+HA2	Flu.B_Phu/.HA1	Flu.B_Phu/.HA1+HA2	Flu.H1N1.HA1	Flu.H1N1.HA1+HA2	Flu.H3N2.HA1	Flu.H3N2.HA1+HA2
SARS.CoV.2.NP	1.00																																				
SARS.CoV.2.S1+S2	0.09	1.00																																			
SARS.CoV.2.S2	0.33	0.53	1.00																																		
SARS.CoV.2.S1.mFcTag	0.03	0.87	0.34	1.00																																	
SARS.CoV.2.S1	0.08	0.82	0.55	0.78	1.00																																
SARS.CoV.2.S1.HisTag	0.08	0.77	0.52	0.79	0.92	1.00																															
SARS.CoV.2.S1.RBD.mFc	0.05	0.84	0.42	0.90	0.87	0.91	1.00																														
SARS.CoV.2.Spike.RBD.rFc	0.06	0.83	0.45	0.89	0.90	0.94	0.96	1.00																													
SARS.CoV.2.Spike.RBD.His.Bac	0.12	0.71	0.56	0.71	0.90	0.91	0.86	0.89	1.00																												
SARS.CoV.2.Spike.RBD.His.HEK	0.11	0.66	0.55	0.67	0.85	0.92	0.81	0.87	0.93	1.00																											
SARS.CoV.NP	0.93	0.07	0.30	0.02	0.06	0.06	0.04	0.04	0.10	0.10	1.00																										
SARS.CoV.S1.HisTag	0.18	0.23	0.48	0.16	0.47	0.41	0.28	0.32	0.52	0.53	0.17	1.00																									
SARS.CoV.S1.RBD.HisTag	0.16	0.36	0.53	0.30	0.62	0.61	0.46	0.51	0.72	0.74	0.14	0.83	1.00																								
SARS.CoV.S1.RBD.rFcTag	0.12	0.47	0.53	0.42	0.72	0.71	0.58	0.64	0.78	0.80	0.10	0.74	0.93	1.00																							
MERS.CoV.NP	0.03	0.01	0.04	0.00	0.01	0.01	0.01	0.01	0.01	0.01	0.04	0.03	0.02	0.02	1.00																						
MERS.CoV.S1.ECD.1-1297.HisTag	0.05	0.22	0.32	0.15	0.24	0.18	0.17	0.18	0.19	0.17	0.05	0.21	0.18	0.19	0.01	1.00																					
MERS.CoV.S1.RBD.367.606.rFcTa	0.00	0.01	0.02	0.00	0.03	0.00	0.00	0.00	0.00	0.00	0.00	0.12	0.03	0.04	0.00	0.15	1.00																				
DcCoV.HKU23.NP	0.00	0.02	0.03	0.01	0.02	0.01	0.01	0.01	0.01	0.01	0.00	0.01	0.01	0.02	0.02	0.07	0.01	1.00																			
hCoV.229E.S1	0.01	0.00	0.01	0.00	0.01	0.01	0.01	0.01	0.01	0.01	0.01	0.01	0.01	0.01	0.01	0.02	0.01	0.07	1.00																		
hCoV.229E.S1_S2	0.01	0.02	0.02	0.01	0.03	0.01	0.01	0.01	0.02	0.01	0.01	0.05	0.03	0.03	0.01	0.09	0.12	0.08	0.82	1.00																	
hCoV.HKU1.NP	0.00	0.02	0.02	0.01	0.02	0.02	0.01	0.02	0.01	0.02	0.00	0.01	0.01	0.02	0.02	0.05	0.01	0.46	0.03	0.04	1.00																
hCoV.HKU1.S1_AA1.760	0.00	0.02	0.02	0.01	0.02	0.01	0.01	0.01	0.01	0.01	0.00	0.01	0.01	0.01	0.01	0.02	0.00	0.10	0.11	0.11	0.13	1.00															
hCoV.HKU1.S1_S2	0.10	0.14	0.22	0.06	0.14	0.09	0.09	0.09	0.13	0.09	0.09	0.14	0.13	0.14	0.03	0.17	0.06	0.13	0.11	0.18	0.09	0.18	1.00														
hCoV.NL63.NP	0.05	0.05	0.06	0.02	0.04	0.03	0.04	0.03	0.04	0.03	0.05	0.03	0.04	0.05	0.01	0.08	0.01	0.18	0.08	0.10	0.10	0.04	0.16	1.00													
hCoV.NL63.S1	0.00	0.01	0.01	0.00	0.01	0.01	0.01	0.01	0.01	0.01	0.00	0.01	0.02	0.02	0.01	0.05	0.02	0.05	0.17	0.15	0.04	0.05	0.08	0.11	1.00												
hCoV.NL63.S1_S2	0.01	0.05	0.04	0.02	0.05	0.03	0.03	0.03	0.03	0.02	0.01	0.04	0.03	0.05	0.02	0.14	0.08	0.08	0.22	0.29	0.04	0.07	0.20	0.19	0.66	1.00											
hCoV.OC43.HE	0.00	0.01	0.02	0.00	0.01	0.01	0.01	0.01	0.01	0.01	0.00	0.01	0.01	0.02	0.01	0.03	0.01	0.10	0.06	0.06	0.05	0.12	0.11	0.03	0.11	0.11	1.00										
hCoV.OC43.NP	0.00	0.01	0.02	0.00	0.02	0.01	0.01	0.01	0.01	0.01	0.00	0.01	0.01	0.02	0.01	0.04	0.01	0.72	0.03	0.03	0.38	0.05	0.08	0.12	0.05	0.05	0.09	1.00									
hCoV.OC43.S1_S2ECD.HisTag	0.11	0.16	0.24	0.08	0.15	0.12	0.11	0.11	0.12	0.11	0.10	0.13	0.12	0.14	0.04	0.31	0.03	0.24	0.10	0.16	0.11	0.15	0.46	0.17	0.09	0.25	0.17	0.15	1.00								
Flu.B_Mal/.HA1	0.00	0.02	0.01	0.01	0.02	0.02	0.03	0.02	0.02	0.01	0.00	0.01	0.01	0.02	0.01	0.04	0.01	0.08	0.06	0.08	0.05	0.05	0.10	0.09	0.10	0.16	0.05	0.06	0.09	1.00							
Flu.B_Mal/.HA1+HA2	0.00	0.04	0.01	0.03	0.04	0.03	0.04	0.04	0.03	0.02	0.00	0.02	0.02	0.04	0.01	0.03	0.02	0.08	0.09	0.14	0.03	0.06	0.15	0.11	0.07	0.19	0.04	0.04	0.13	0.68	1.00						
Flu.B_Phu/.HA1	0.00	0.03	0.01	0.02	0.03	0.03	0.03	0.03	0.03	0.03	0.00	0.02	0.03	0.04	0.02	0.04	0.01	0.10	0.07	0.08	0.08	0.07	0.10	0.08	0.10	0.15	0.08	0.08	0.13	0.40	0.38	1.00					
Flu.B_Phu/.HA1+HA2	0.00	0.04	0.01	0.03	0.02	0.01	0.02	0.02	0.01	0.01	0.00	0.01	0.01	0.01	0.01	0.02	0.00	0.05	0.05	0.06	0.02	0.04	0.12	0.07	0.02	0.09	0.03	0.02	0.10	0.19	0.41	0.35	1.00				
Flu.H1N1.HA1	0.01	0.02	0.03	0.01	0.03	0.02	0.02	0.02	0.03	0.02	0.01	0.03	0.03	0.03	0.01	0.02	0.00	0.04	0.04	0.04	0.03	0.02	0.07	0.06	0.04	0.08	0.02	0.03	0.06	0.09	0.15	0.17	0.13	1.00			
Flu.H1N1.HA1+HA2	0.02	0.02	0.03	0.01	0.03	0.02	0.02	0.02	0.03	0.02	0.02	0.02	0.03	0.03	0.02	0.02	0.00	0.04	0.04	0.05	0.02	0.02	0.09	0.07	0.03	0.08	0.02	0.01	0.09	0.10	0.18	0.16	0.21	0.81	1.00		
Flu.H3N2.HA1	0.00	0.02	0.01	0.00	0.02	0.01	0.01	0.01	0.01	0.01	0.00	0.01	0.02	0.03	0.01	0.03	0.01	0.08	0.03	0.05	0.08	0.03	0.09	0.07	0.05	0.10	0.04	0.08	0.09	0.18	0.19	0.19	0.11	0.15	0.12	1.00	
Flu.H3N2.HA1+HA2	0.01	0.04	0.02	0.03	0.02	0.01	0.02	0.01	0.01	0.01	0.00	0.01	0.01	0.01	0.00	0.01	0.00	0.02	0.01	0.03	0.00	0.01	0.10	0.05	0.00	0.04	0.01	0.01	0.08	0.08	0.22	0.08	0.48	0.10	0.19	0.26	1.00

Supplementary table 4

Santa Ana Cohort Demographics

	Overall	Reactive	Non-Reactive
**Overall**	N=3218	852 (27%)	2342 (73%)
**Age**			
Child (5-17)	390 (12%)	108 (28%)	278 (71%)
Adult (18+)	2824 (88%)	744 (26%)	2060 (73%)
**Gender**			
Male	1239 (39%)	306 (25%)	925 (75%)
Female	1971 (61%)	545 (28%)	1410 (72%)
**Race/Ethnicity**			
Non-Hispanic White	274 (9%)	32 (12%)	240 (88%)
Hispanic	27333 (85%)	791 (29%)	1921 (70%)
Asian	144 (5%)	21 (15%)	122 (85%)
Other non-Hispanic	52 (2%)	3 (6%)	49 (94%)

Health Care Workers Cohort Demographics

Variable	Total N	20-May	20-Dec	21-Jan	21-Feb
# samples	2420	1060	313	140	750
Gender					
**Female**	1127 (61.1%)	478 (65.3%)	192 (64.2%)	78 (64.5%)	377 (62.3%)
**Male**	554 (30%)	253 (34.6%)	106 (35.5%)	43 (35.5%)	152 (25.1%)
**Other***	2 (0.1%)	1 (0.1%)	1 (0.3%)	0	0
**Not reported**	163 (8.8%)	0	0	0	76 (12.6%)
Race/ethnicity					
**Asian**	623 (33.7%)	239 (32.7%)	108 (36.1%)	50 (41.3%)	225 (37.2%)
**White**	539 (29.2%)	237 (32.4%)	122 (40.8%)	42 (34.7%)	137 (22.6%)
**Latino**	347 (18.8%)	185 (25.3%)	42 (14%)	23 (19%)	97 (16%)
**Other***	149 (8.1%)	62 (8.5%)	20 (6.7%)	6 (5%)	61 (10.1%)
**Not reported**	188 (10.2%)	9 (1.2%)	7 (2.3%)	0	85 (14%)
Age					
**18-34 years**	702 (38%)	263 (35.9%)	130 (43.5%)	65 (53.7%)	244 (40.3%)
**35-54 years**	758 (41.1%)	363 (49.6%)	122 (40.8%)	38 (31.4%)	233 (38.5%)
**55+ years**	219 (11.9%)	103 (14.1%)	47 (15.7%)	18 (14.9%)	51 (8.4%)
**Not reported**	167 (9%)	3 (0.4%)	0	0	77 (12.7%)
COVID symptoms (any)					
**No**	1005 (43.2%)	383 (52.3%)	178 (59.5%)	85 (70.2%)	359 (59.3%)
**Yes**	678 (29.2%)	349 (47.7%)	121 (40.5%)	36 (29.8%)	170 (28.1%)
**Not reported**	163 (7%)	0	0	0	76 (12.6%)
Vaccinated					
**No**	1299 (70.4%)	732 (100%)	298 (99.7%)	35 (28.9%)	145 (24%)
**Yes**	547 (29.6%)	0	1 (0.3%)	86 (71.1%)	460 (76%)
