# Supplementary material for: Distinct SARS-CoV-2 antibody reactivity patterns elicited by natural infection and mRNA vaccination
Source: NPJ Vaccines. 2021 Nov 4;6:132. doi: 10.1038/s41541-021-00396-3 (PMC8568980; doi:10.1038/s41541-021-00396-3)
Supplement: Supplementary file 2 — Reporting summary [file 41541_2021_396_MOESM2_ESM.pdf]

nature portfolio

Corresponding author(s): Rafael Ramiro de Assis

Last updated by author(s): Sep 9, 2021

Reporting Summary

Nature Portfolio wishes to improve the reproducibility of the work that we publish. This form provides structure for consistency and transparency in reporting. For further information on Nature Portfolio policies, see our Editorial Policies and the Editorial Policy Checklist.

Statistics

For all statistical analyses, confirm that the following items are present in the figure legend, table legend, main text, or Methods section.

n/a	Confirmed	
☐	☒	The exact sample size (*n*) for each experimental group/condition, given as a discrete number and unit of measurement
☐	☒	A statement on whether measurements were taken from distinct samples or whether the same sample was measured repeatedly
☐	☒	The statistical test(s) used AND whether they are one- or two-sided<br>*Only common tests should be described solely by name; describe more complex techniques in the Methods section.*
☒	☐	A description of all covariates tested
☐	☒	A description of any assumptions or corrections, such as tests of normality and adjustment for multiple comparisons
☐	☒	A full description of the statistical parameters including central tendency (e.g. means) or other basic estimates (e.g. regression coefficient) AND variation (e.g. standard deviation) or associated estimates of uncertainty (e.g. confidence intervals)
☒	☐	For null hypothesis testing, the test statistic (e.g. *F*, *t*, *r*) with confidence intervals, effect sizes, degrees of freedom and *P* value noted<br>*Give P values as exact values whenever suitable.*
☒	☐	For Bayesian analysis, information on the choice of priors and Markov chain Monte Carlo settings
☐	☒	For hierarchical and complex designs, identification of the appropriate level for tests and full reporting of outcomes
☒	☐	Estimates of effect sizes (e.g. Cohen's *d*, Pearson's *r*), indicating how they were calculated

Our web collection on statistics for biologists contains articles on many of the points above.

Software and code

Policy information about availability of computer code

Data collection

Data was collected using the software ScanArray Express (PerkinElmer) version 3.0

Data analysis

The Construction of the prediction models was performed as following.
1. Data is pre-processed and normalized as described above.
2. The reference data set was decomposed into a vector using the function ‘unmatrix’ from the package gData (version 2.18.0).
3. A mixture model is calculated for the vector using the function ‘normalmixEM’ from the package ‘mixtools’ (version 1.2.0).
4. A cutoff is then calculated as 3 standard deviations over the mean of the negative signal curve.
5. Wilcox test for each antigen was performed comparing the positive controls and negatives control, considering significant, antigens with $p < 0.05$.

The selection was performed as follows:
1. For every possible combination of the seropositive SARS-CoV-2 antigens from 1 all, the reference set was randomly divided into a training and a testing sets at a 70%/30% ratio.
2. A logistic regression was generated using the reference set. The regression was generated using the function ‘glm’ of the ‘stats’ package (version 4.0.0).and a ROC curve was calculated (package pROC version 1.16.2).
3. The optimal coordinates of the ROC curve were obtained based on the ‘youden index’, by prioritizing the specificity.
4. The coordinates were obtainded using the function ‘coords’ from the pROC library. The coordinates are obtainded in a table format with each row containing a regression threshold and its related specificity and sensitivity.
5. The coordinates were then subset to represent specificities of 0.95 or higher. A threshold was then defined as the threshold on the coordinate with the highest specificity on the subset.
6. A logistic regression was then calculated using the testing set and each sample classified as negative or positive by comparison with the threshold.
7. A confusion matrix was calculated by comparing the predicted outcomes and the known classifications (“known negative” or “Known positive”) and the prediction specificity and sensitivity stored into a vector.

8. This analysis was repeated 1000 times and the sensitivity and sensitivity calculated as the mean predicted performance of all repetitions.

The performance outcome for each antigen combination was analyzed and a selection of the best performing combinations was made based on the specificity and sensitivity. The selected candidates were then tested using the full reference sample set. The test was performed as follows:
1. A logistic regression for each antigen combination candidate using the full reference set. Then a ROC curve was calculated and the coordinate table with all curve points was obtained.
2. The coordinates of each candidate were compared in order to select the candidate with the highest sensitivity, given a fixed specificity of 1 (100%).

For manuscripts utilizing custom algorithms or software that are central to the research but not yet described in published literature, software must be made available to editors and reviewers. We strongly encourage code deposition in a community repository (e.g. GitHub). See the Nature Portfolio guidelines for submitting code & software for further information.

Data

Policy information about availability of data

All manuscripts must include a data availability statement. This statement should provide the following information, where applicable:

- Accession codes, unique identifiers, or web links for publicly available datasets
- A description of any restrictions on data availability
- For clinical datasets or third party data, please ensure that the statement adheres to our policy

The raw data that support the findings of this study are available from the corresponding author upon reasonable request.

Field-specific reporting

Please select the one below that is the best fit for your research. If you are not sure, read the appropriate sections before making your selection.

☒ Life sciences ☐ Behavioural & social sciences ☐ Ecological, evolutionary & environmental sciences

For a reference copy of the document with all sections, see nature.com/documents/nr-reporting-summary-flat.pdf

Life sciences study design

All studies must disclose on these points even when the disclosure is negative.

Sample size	From the University of California Irvine Medical Center (UCIMC, Orange County, CA, USA), samples from 949 individuals were collected at several time points. From residents of the Orange County community, 3347 individuals and from the UCIMC biorepository, a collection of 563 longitudinal plasma specimens from 86 individuals was used.
Data exclusions	No data was excluded.
Replication	The replicates here mentioned are in reference to the replicates of the spots printed on the arrays. All replicates were detected and used to calculate the mean fluorescence intensity (MFI) of each printed antigen. Due to the nature and underlying costs of the microarray assay, technical replicates for the probing experiments were not performed.
Randomization	No randomization was used on the allocation of samples. The additional samples were added based on availability.
Blinding	The analysis of the array data was performed based on non subjective pre determined parameters. Samples were provided by collaborators and groups built independently of each other so, no bias could be generated by non blinded data analysis.

Reporting for specific materials, systems and methods

We require information from authors about some types of materials, experimental systems and methods used in many studies. Here, indicate whether each material, system or method listed is relevant to your study. If you are not sure if a list item applies to your research, read the appropriate section before selecting a response.

Materials & experimental systems

n/a	Involved in the study	
☐	☒	Antibodies
☒	☐	Eukaryotic cell lines
☒	☐	Palaeontology and archaeology
☒	☐	Animals and other organisms
☐	☒	Human research participants
☒	☐	Clinical data
☒	☐	Dual use research of concern

Methods

n/a	Involved in the study	
☒	☐	ChIP-seq
☒	☐	Flow cytometry
☒	☐	MRI-based neuroimaging

Antibodies

Antibodies used: Goat Anti-Human IgG Fc conjugated to Quantum dot 800, GraceBio, SKU 110610; Goat F(ab')2 Anti-Human IgM conjugated to Quantum dot 655, GraceBio, SKU 110630 ; Goat anti-human IgA conjugated to Quantum dot 655, GraceBio, SKU 110620.

Validation: https://onlinelibrary.wiley.com/doi/epdf/10.1002/pmic.201700277

Human research participants

Policy information about studies involving human research participants

Population characteristics: Orange County participant were 12% from 15 to 17, and 88% over 18 years old. 39% female and 61% male. 9% reported as being Non-Hispanic White, 85% Hispanic, 5% Asian and 2% Other Non-Hispanic. The HCW group was, as reported in December 2020, 62.3% female, 25.1% male and 12.6% not reported. 37.2% reported as Asian, 22.6% White, 16% Latino, 10.1% as Other and 14% Not reported. The age group age was 40%. beteen 18 and 34 years, 38.5% between 25 and 54, 8.4% over 55 and 12.7% did not report their age.

Recruitment: For the HCW group, participants were invited as described at https://www.medrxiv.org/content/10.1101/2020.12.17.20248430v1. Orange County population recruitment was performed as described at https://www.ncbi.nlm.nih.gov/pmc/articles/PMC7862219/. All samples were de-identified.

Ethics oversight: UCI Institutional Review Board (HS# 2020-5952) and (HS# 2020-5818), Comprehensive Clinical, Imaging and Histological Database for the Study of COVID-19 Infection and Outcomes (HS# 2020-5783).

Note that full information on the approval of the study protocol must also be provided in the manuscript.
